# Subgroup-specific prognostic signaling and metabolic pathways in pediatric medulloblastoma

**DOI:** 10.1186/s12885-019-5742-x

**Published:** 2019-06-11

**Authors:** Ae Kyung Park, Ji Yeoun Lee, Heesun Cheong, Vijay Ramaswamy, Sung-Hye Park, Marcel Kool, Ji Hoon Phi, Seung Ah Choi, Florence Cavalli, Michael D. Taylor, Seung-Ki Kim

**Affiliations:** 1 College of Pharmacy and Research Institute of Life and Pharmaceutical Sciences, Sunchon National University, Suncheon, Korea; 2 Division of Pediatric Neurosurgery, Seoul National University Children’s Hospital, Department of Neurosurgery, Seoul National University College of Medicine, Seoul, Korea; 3 Department of Anatomy, Neural Development and Anomaly Lab, Seoul National University College of Medicine, Seoul, Korea; 4 Neuroscience Research Institute, Seoul National University College of Medicine, Seoul, Korea; 5 Division of Cancer Biology, National Cancer Center, Goyang, Korea; 6 Division of Haematology/Oncology, The Hospital for Sick Children, Toronto, Canada; 7 Department of Pathology, Seoul National University Hospital, Seoul National University College of Medicine, Seoul, Korea; 8 Division of Paediatric Neurooncology, German Cancer Research Center (DKFZ), Heidelberg, Germany; 9 Programme in Developmental and Stem Cell Biology, Hospital for Sick Children, Toronto, Canada; 10 Department of Laboratory Medicine and Pathobiology, University of Toronto, Toronto, Canada

**Keywords:** Medulloblastoma, Subgroup, Prognosis, Signaling pathway, Metabolic pathway, Targeted therapy

## Abstract

**Background:**

Using a pathway-focused approach, we aimed to provide a subgroup-specific basis for finding novel therapeutic strategies and further refinement of the risk stratification in pediatric medulloblastoma.

**Method:**

Based on genome-wide Cox regression and Gene Set Enrichment Analysis, we investigated prognosis-related signaling pathways and core genes in pediatric medulloblastoma subgroups using 530 patient data from Medulloblastoma Advanced Genomic International Consortium (MAGIC) project. We further examined the relationship between expression of the prognostic core genes and frequent chromosome aberrations using broad range copy number change data.

**Results:**

In SHH subgroup, relatively high expression of the core genes involved in p53, PLK1, FOXM1, and Aurora B signaling pathways are associated with poor prognosis, and their average expression synergistically increases with co-occurrence of losses of 17p, 14q, or 10q, or gain of 17q. In Group 3, in addition to high MYC expression, relatively elevated expression of PDGFRA, IGF1R, and FGF2 and their downstream genes in PI3K/AKT and MAPK/ERK pathways are related to poor survival outcome, and their average expression is increased with the presence of isochromosome 17q [i(17q)] and synergistically down-regulated with simultaneous losses of 16p, 8q, or 4q. In Group 4, up-regulation of the genes encoding various immune receptors and those involved in NOTCH, NF-κB, PI3K/AKT, or RHOA signaling pathways are associated with worse prognosis. Additionally, the expressions of Notch genes correlate with those of the prognostic immune receptors. Besides the Group 4 patients with previously known prognostic aberration, loss of chromosome 11, those with loss of 8q but without i(17q) show excellent survival outcomes and low average expression of the prognostic core genes whereas those harboring 10q loss, 1q gain, or 12q gain accompanied by i(17q) show bad outcomes. Finally, several metabolic pathways known to be reprogrammed in cancer cells are detected as prognostic pathways including glutamate metabolism in SHH subgroup, pentose phosphate pathway and TCA cycle in Group 3, and folate-mediated one carbon-metabolism in Group 4.

**Conclusions:**

The results underscore several subgroup-specific pathways for potential therapeutic interventions: SHH-GLI-FOXM1 pathway in SHH subgroup, receptor tyrosine kinases and their downstream pathways in Group 3, and immune and inflammatory pathways in Group 4.

**Electronic supplementary material:**

The online version of this article (10.1186/s12885-019-5742-x) contains supplementary material, which is available to authorized users.

## Background

With advancements in molecular genomics, many aspects of the genetic basis of tumorigenesis in various cancers have been clarified. Brain tumors have also been a topic of genomic evaluation, and medulloblastoma is one of the most actively studied entities. Consensus has been reached that there are at least 4 subgroups of medulloblastoma: WNT, SHH, Group 3, and Group 4. [1–4]. In the recent update of the 2016 World Health Organization Classification of Tumors of the Central Nervous System (2016 CNS WHO), the subclassification has been incorporated into the diagnosis of medulloblastoma. Furthermore, preliminary studies have focused on the development of new prognostic biomarkers, novel stratification strategies, or targeted therapies for each subgroup and the elucidation of the subgroup-specific dysregulation of signaling pathways, cytogenetic aberrations, and epigenetic deregulation [2, 5–8]. However, considerable heterogeneity has remained within the subgroups, among which differences in prognosis are the most clinically important. Combinations of clinical features and several genetic, chromosomal markers have been suggested to classify risk groups within the four subgroups. Recent studies based on integrated multi-omics data including DNA methylation profiles have led to identify refined subtypes within each subgroup of medulloblastoma [9, 10]. The newly defined subtypes were enriched with distinctive clinical and genomic features and several new subtypes were associated with relatively favorable or worse prognosis. Hence, we aimed to discover the biological mechanisms underlying differential clinical outcomes within medulloblastoma subgroups by employing subgroup-specific Gene Set Enrichment Analysis (GSEA) combined with disease progression data analysis [11]. Through the identification of signaling pathways that reflect poor clinical outcome subgroup specifically, we hope to develop new candidates for biomarkers or therapeutic targets for application in precision medicine.

## Methods

Total 763 expression data of primary medulloblastomas collected via Medulloblastoma Advanced Genomic International Consortium (MAGIC) in a previous study was downloaded from Gene Expression Omnibus (GSE85217) [9]. The raw cel files were preprocessed using the Robust Multi-array Average algorithm [12] and log_2_ transformed. To remove multiple probe sets for a given gene in the microarray data, the probe set with the largest inter-quartile range across the samples was selected as a representative one. Sample information including clinical data, subgroup and subtype information, and broad range copy number change data was obtained from the previous study [9]. We further selected only the samples from the patients with age < 18 and with available overall survival information. The final dataset comprised of 530 samples including WNT (*n* = 49), SHH (*n* = 121), Group 3 (*n* = 107), and Group 4 (*n* = 253). Subsequently, the expression values of each gene were rescaled to relative expression values across the 530 samples ranging from 0 to 1. The 530 samples were divided into exploratory (70% of samples) and validation (30% of samples) datasets, based on randomized sampling stratified by subgroup and 2-year survival status (dead, alive, or censored). Using the exploratory dataset, survival analysis was performed to assess the prognostic impact of individual genes on overall survival in WNT subgroup (*n* = 34), SHH subgroup (*n* = 85), Group 3 (*n* = 74), and Group 4 (*n* = 177). More specifically, genome-wide univariable Cox regression analysis was employed to calculate proportional hazard ratios (HRs) that indicate the effects of one unit increases in relative expressions of individual genes on risk of death. In this survival analysis step, only the genes with high expression (log_2_ maximum expression ≥7) and large variation (log_2_ expression range ≥ 1.5) were analyzed in each subgroup: the final numbers of the genes analyzed were 7832 for WNT, 13,059 for SHH, 12,779 for Group 3, and 13,076 for Group 4. However, in subsequent analyses, WNT subgroup was omitted due to the very low frequency of death event (3 out of 49) which was not suitable for application of survival analysis. To test the fundamental assumption of proportional hazards of the Cox model, we assessed goodness-of-fit for proportional hazards model [13] and found that the assumption is reasonable for most of the genes investigated (94.9% in SHH subgroup, 96.2% in Group 3, 98.8% in Group 4) (Additional file 1: Table S1). Then, GSEA was performed to detect overrepresented canonical pathways associated with poor prognosis based on curated gene sets of MSigDB (C2) with 1000 non-parametric permutations using pre-ranked genes sorted by the natural log-transformed HRs obtained from the Cox regression analysis [14]. The analysis was confined to total 988 gene sets of size ≤50 to avoid too broad pathways. Initial enriched pathways were detected at a nominal *p*-value < 0.05 in positive and negative directions respectively, and the genes in the leading-edge subset were considered as core genes in each pathway [14]. Many gene sets were identified redundantly since the gene sets of canonical pathways (C2) in GSEA were collected from multiple databases [14]. Accordingly, we focused on the results from three representative databases, Pathway Interaction Database (PID), Kyoto Encyclopedia of Genes and Genomes (KEGG), and BioCarta (BIOCARTA). Then, collective prognostic power of each pathway was evaluated in both exploratory and validation datasets using Cox regression analysis with average relative expression values of the core genes. To further test the robustness of prognostic significance of each pathway, 1000 bootstrap datasets were generated from entire samples by simple random sampling with replacement. Cox regression model was run on each of the bootstrap samples and bootstrap value for each pathway was calculated by the percentage of bootstrap *p*-values less than 0.05. We also investigated frequently observed chromosome aberrations that were significantly associated with prognosis in each subgroup. Using broad range copy number change data, we applied univariable Cox regression analysis to detect subgroup-specific prognosis-related gain or loss of each chromosome arm that occurred in each subgroup with a high frequency (≥ 10%). According to the estimated HR, the direction of chromosome aberration is designated “risk” (HR > 1) or “protective” (HR < 1). All data analyses were performed using R statistical software (http://www.R-project.org/).

## Results

Ten year Kaplan-Meier plots based on 530 overall survival data of pediatric medulloblastoma patients revealed that survival prognoses of the four subgroups were largely congruent with previous studies in both exploratory and validation datasets [2, 5, 8]. The WNT subgroup was associated with the best survival, SHH subgroup and Group 4 with intermediate survival, and Group 3 with the poorest survival (Additional file 2: Figure S1).

### SHH subgroup

**Fig. 1:**
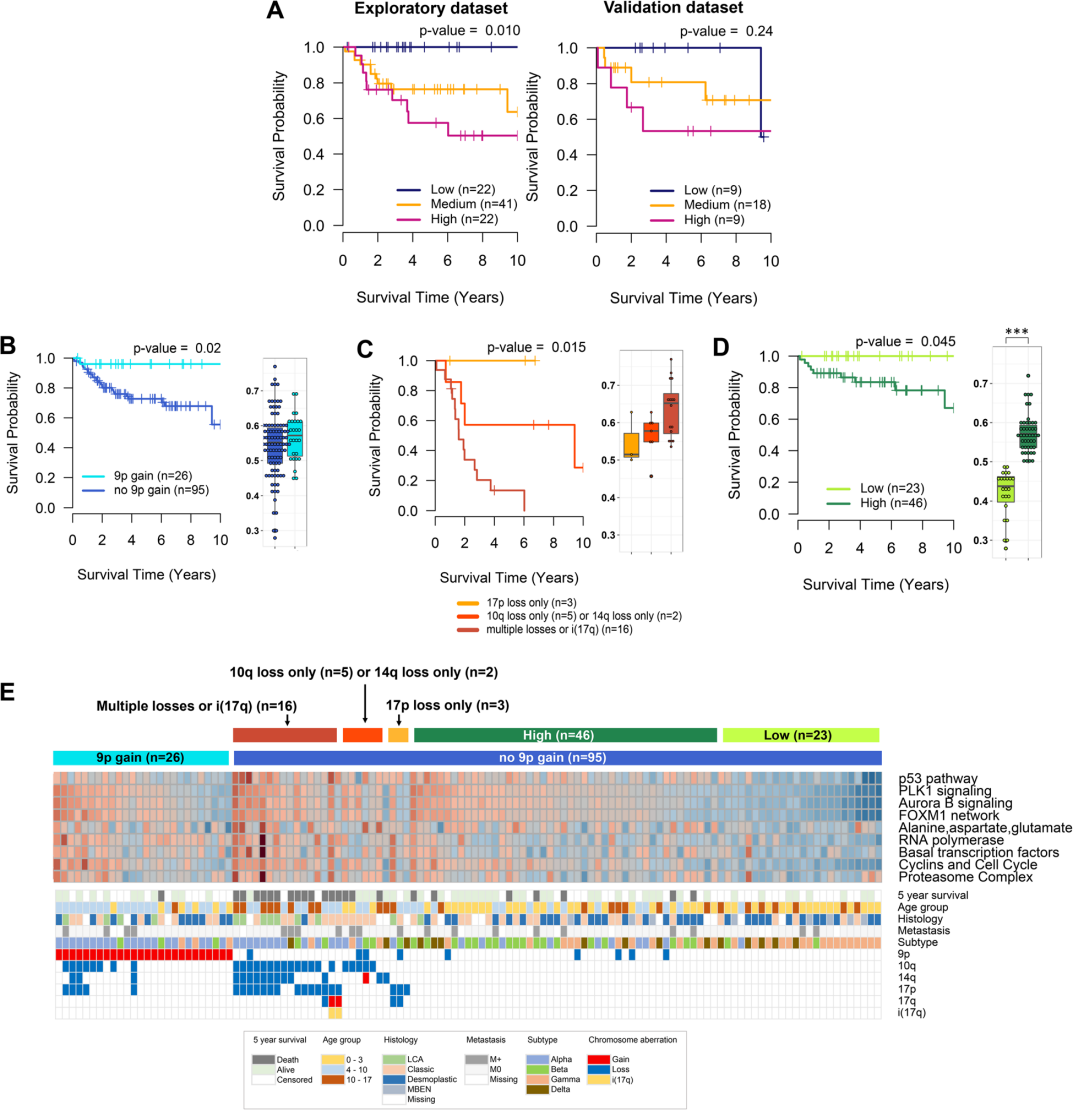
Prognostic power of core genes identified in SHH subgroup. **a**, Kaplan-Meier curves and log-rank tests of the three groups stratified by Q1 (25% quantile) and Q3 (75% quantile) of the average expression of 31 core genes from top two PID pathways. **b**, Kaplan-Meier curve with log-rank test and boxplot of the average expression of 31 core genes of the two groups divided by the status of 9p gain. **c**, Kaplan-Meier curve with log-rank test and boxplot of the average expression of 31 core genes of the three groups of 26 patients who harbor i(17q) or losses of 10q, 14q, or 17p but not 9p gain, stratified by the status of i(17q) and losses of 10q, 14q, and 17p. **d**, Kaplan-Meier curve with log-rank test and boxplot of the average expression of 31 core genes of the two groups of 69 patients who do not harbor 9p gain, i(17q), and losses of 10q, 14q, or 17p, stratified by the average expression of 31 core genes. **e**, Heatmap of expression of core genes from the nine prognostic canonical pathways with clinical information, prognostic chromosome aberrations, and subtype [9]. ***, Mann–Whitney U test *p*-value < 0.001

In GSEA analysis, nine canonical pathways in SHH subgroup are detected to be prognostic toward positive direction including four PID canonical pathways, “p53 pathway”, “PLK1 signaling events”, “Aurora B signaling”, and “ FOXM1 transcription factor network”, three KEGG pathways, “Alanine, aspartate, and glutamate metabolism”, “RNA polymerase”, and “Basal transcription factors”, and two BIOCARTA pathways, “Cyclins and Cell Cycle Regulation” and “Proteasome complex” (Table 1, Additional file 1: Table S2). In bootstrap analysis with 1000 replications, eight out of nine canonical pathways show statistically significant results in more than 90% of resampled datasets. According to the average expression values of 31 core genes from the top two PID canonical pathways (“p53 pathway” and “PLK1 signaling events”), the patients are separated into three different survival groups similarly in both exploratory and validation datasets (Fig. 1a). Furthermore, we also obtain statically significant results with a whole dataset (*n* = 121) in log-rank tests with average expression of core genes in nine canonical pathways respectively (Additional file 1: Table S2).

**Table 1:** Top five prognosis-related canonical pathways in SHH subgroup

Database	Name of gene set (GSEA)	Exploratory dataset (*n* = 85)		Validation dataset (*n* = 36)		1000 bootstrap datasets (n = 121)	Representative core genes**
		HR*	*p*-value	HR*	*p*-value	% of *p*-value < 0.05	
PID	p53 pathway	4.59	< 0.0001	3.19	0.127	100.0	TRIM28,CCNA2,ABL1,SETD7,CSE1L,HIPK2,RPL11
	PLK1 signaling events	2.02	0.0035	1.72	0.093	95.6	NUDC,PLK1,AURKA,TPX2,BUB1,CDC20,TPT1, BORA,CENPE,KIF20A,PRC1,CCNB1,CDC25C,WEE1,ECT2
	Aurora B signaling	1.73	0.0155	1.84	0.086	91.9	CDCA8,SMC2,AURKA,KIF2C,BUB1,RACGAP1, BIRC5,NCAPD2,KIF23,KIF20A,NCAPH
	FOXM1 transcription factor network	1.89	0.0119	1.75	0.109	90.8	PLK1,CCNA2,FOXM1,BIRC5,NEK2,ETV5, CCNB1,CENPF,BRCA2,CDK4,CHEK2,CKS1B
KEGG	Alanine aspartate and glutamate metabolism	4.85	0.0001	1.76	0.432	94.3	PPAT,GLUD1,GPT2
	RNA polymerase	1.96	0.0032	2.36	0.022	93.3	POLR2E,POLR3H,POLR2B,POLR2J,POLR2D, POLR3K,POLR1B,POLR1A
	Basal transcription factors	2.48	0.0002	1.49	0.580	91.6	GTF2F1,TAF4,GTF2A1L,TAF11
BIOCARTA	Cyclins and Cell Cycle Regulation	1.92	0.0127	3.17	0.029	91.7	CCNA1,CDKN2C,CCNB1,CDC25A,TFDP1,CDK4
	Proteasome Complex	1.71	0.0053	1.57	0.274	79.1	PSMB7,PSMA7,PSMD14,PSMB2

* Hazard ratio per 0.1 increment in average relative expression of core genes

** Representative core genes are significantly associated with overall survival (p-value < 0.05) in Cox regression analysis with all samples

In association analyses between survival outcomes and copy number changes, gain of 9p is detected as a significant protective aberration (Additional file 1: Table S3). The patients with 9p gain show excellent prognosis (Fig. 1b), however, the expression of 31 prognostic core genes is not significantly low in the tumors with 9p gain (Fig. 1b boxplot). On the other hand, losses of 17p, 14q, 10q, and isochromosome 17q [i(17q)] are observed to be risk aberrations (Additional file 1: Table S3). After excluding the patients with 9p gain, we further find that 16 patients harboring i(17q) or more than two losses out of 10q, 14q, and 17p show the worst survival outcome (Fig. 1c). Seven patients with either one of 10q loss or 14q loss show intermediate survival rates while three patients with 17p loss alone show the best prognosis. Furthermore, the highest expression level of 31 prognostic core genes is observed in the 16 patients with i(17q) or multiples losses of 10q, 14q, or 17p whereas the lowest is detected in the three patients with 17p loss alone (Fig. 1c boxplot). Finally, 69 patients who do not possess any risk or protective aberrations can be divided into two different survival groups according to the average expression of the 31 core genes (Fig. 1d). The average expressions of the core genes from the nine prognostic canonical pathways is presented in a heatmap including clinical information, subtype information recently reported by Cavalli et al. [9], and the stratification of patients according to the status of prognostic chromosome aberrations and gene expressions shown in Fig. 1b-d (Fig. 1e). The heatmap shows that the average expressions of the core genes of the nine pathways considerably correlate with one another. On the other hand, the average expression of the 31 core genes from the top two PID canonical pathways is also significantly associated with subtype, age group, and histology; the highest expression is observed in subtype of SHH α and histological group of large cell/anaplastic (LCA) (Additional file 2: Figure S2A).

### Group 3

**Fig. 2:**
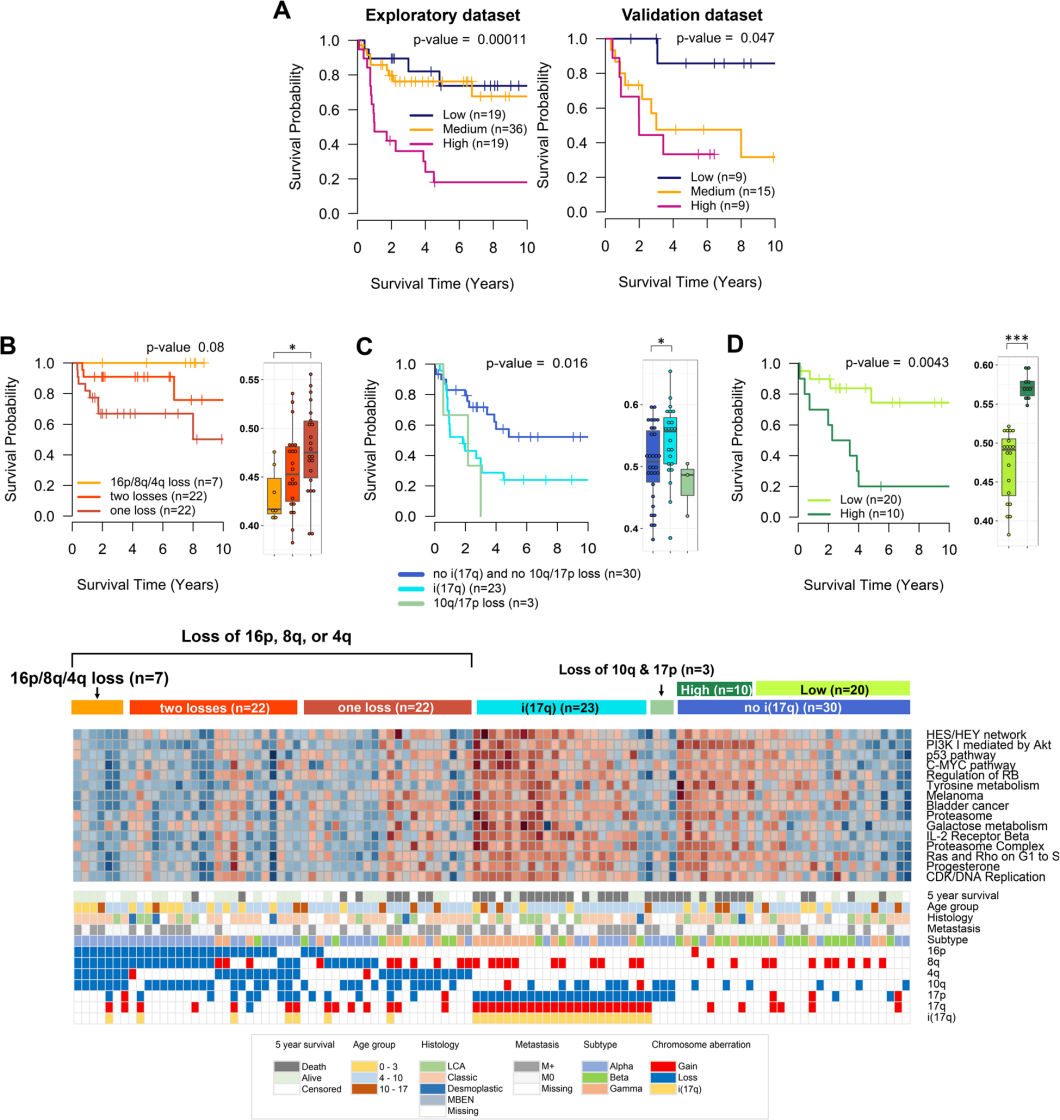
Prognostic power of the core genes identified in Group 3. **a**, Kaplan-Meier curves and log-rank tests of the three groups stratified by Q1 (25% quantile) and Q3 (75% quantile) of the average expression of 20 core genes from top two PID pathways. **b**, Kaplan-Meier curve with log-rank test and boxplot of the average expression of 20 core genes of the three groups of 51 patients who harbor losses of 16p, 8q, or 4q, stratified by the number of simultaneous losses of 16p, 8q, and 4q. **c**, Kaplan-Meier curve with log-rank test and boxplot of the average expression of 20 core genes of the two groups of 56 patients who do not harbor losses of 16p, 8q, and 4q, stratified by status of i(17q) and simultaneous loss of 17p and 10q. **d**, Kaplan-Meier curve with log-rank test and boxplot of the average expression of 20 core genes of the two groups of 33 patients who do not harbor i(17q), simultaneous loss of 17p and 10q, and losses of 16p, 8q, and 4q, stratified by the average expression of 20 core genes. E, Heatmap of expression of core genes from the fifteen prognostic canonical pathways with clinical information, prognostic chromosome aberrations, and subtype [9]. *, Mann–Whitney U test *p*-value < 0.05; **, *p*-value < 0.01; ***, *p*-value < 0.001

Total 35 canonical pathways in PID, KEGG, and BIOCARTA are associated with poor prognosis in Group 3 (Additional file 1: Table S2). Of the 35 canonical pathways, 16 pathways show > 90% of statistical significance in 1000 bootstrap samples, including many cancer-related signaling pathways such as “Notch-mediated HES/HEY network” (Notch network), “Class I PI3K signaling events mediated by Akt” (PI3K/Akt), “p53 pathway”, “Melanoma”, “Bladder cancer”, “Proteasome”, and “Influence of Ras and Rho proteins on G1 to S Transition” (Ras/Rho on G1 to S). In addition, the prognostic canonical pathways include several metabolic pathways such as “Tyrosine and galactose metabolism”, “Pentose phosphate pathway”, and “TCA cycle”. Top 5 prognostic canonical pathways in each database ranked by bootstrap percentage are presented with representative prognostic core genes in Table 2. According to the average expression of 20 core genes from the top two PID canonical pathways (“Notch network” and “PI3K/Akt”), low and high expression groups of the Group 3 patients are clearly separated into different survival groups even though medium groups are inconsistently segregated into the low or high group in exploratory and validation datasets respectively (Fig. 2a).

**Table 2:** Top five prognosis-related canonical pathways in Group 3

Database	Name of gene set (GSEA)	Exploratory dataset (*n* = 74)		Validation dataset (*n* = 33)		1000 bootstrap datasets (*n* = 107)	Representative core genes**
		HR*	*p*-value	HR*	*p*-value	% of *p*-value < 0.05	
PID	Notch-mediated HES/HEY network	2.71	0.0001	2.21	0.087	98.8	MYOD1,KDM1A,GAA,MYB,CTBP1
	Class I PI3K signaling events mediated by Akt	2.08	0.0044	2.40	0.021	97.1	YWHAZ,PRKDC,CDKN1B
	p53 pathway	2.15	0.0052	1.79	0.071	92.8	DYRK2,RPL23,PPM1D,RPL11
	C-MYC pathway	1.69	0.0087	2.26	0.040	89.4	RUVBL1,ACTL6A,RUVBL2
	Regulation of retinoblastoma protein	2.01	0.0094	2.23	0.146	89.0	MYOD1,E2F3,CDKN1B,CDK4,CTBP1
KEGG	Tyrosine metabolism	3.40	0.0002	5.22	0.000	100.0	COMT,MIF,GOT2
	Melanoma	4.86	0.0001	4.64	0.048	99.5	FGF2,E2F3,CDK4
	Bladder cancer	2.57	0.0040	5.73	0.020	97.2	MYC,E2F3,CDK4
	Proteasome	1.94	0.0014	1.94	0.031	96.9	PSMB3,PSMC3,PSMB1,PSMC5,PSMD3, PSMA5,PSMB7,PSMA4,PSMD12,PSMA6,PSMD14
	Galactose metabolism	2.13	0.0037	2.50	0.009	95.2	GLA,GAA,PGM2
BIOCARTA	IL-2 Receptor Beta Chain in T cell Activation	3.60	0.0002	6.49	0.009	99.9	PPIA,STAT5B,MYC
	Proteasome Complex	1.82	0.0041	1.67	0.038	95.5	PSMB3,PSMC3,PSMB1,RPN1,PSMA5,PSMB7, PSMA4,PSMD12,PSMA6,PSMD14
	Influence of Ras and Rho proteins on G1 to S	1.87	0.0216	4.20	0.012	92.1	CDKN1B,CDK4
	How Progesterone Initiates Oocyte Membrane	1.99	0.0183	3.66	0.041	86.4	ARPC3
	CDK Regulation of DNA Replication	1.31	0.0418	1.42	0.173	74.6	MCM2,MCM5,ORC6,MCM4,CDT1,CDKN1B

*Hazard ratio per 0.1 increment in average relative expression of core genes

** Representative core genes are significantly associated with overall survival (p-value < 0.05) in Cox regression analysis with all samples

Several chromosome aberrations including losses of 16p, 8q, 16q, 4q, 22q, and 11q are detected as protective aberrations (Additional file 1: Table S4). However, the protective aberrations are significantly associated with one another and many patients possess multiple protective aberrations simultaneously. Thus, we applied multivariable Cox regression analysis and selected the losses of 16p, 8q and 4q as the final protective aberrations because the losses of 16q, 22q, and 11q contributed little to increase in R-squared value. When 51 patients possessing one of the protective aberrations, losses of 16p, 8q and 4q, are divided into three groups according to the number of the losses, seven patients harboring all the losses show the most favorable prognosis and the survival rates are reduced as the number of losses decrease (Fig. 2b). Besides, the number of losses inversely correlates with the average expression levels of the 20 prognostic core genes from the top two PID pathways (Fig. 2b, boxplot). On the other hand, 17p loss and i(17q) are detected as risk aberrations (Additional file 1: Table S4). In multivariable Cox regression analysis, 17p loss is no longer significant when the status of i(17q) is added to the model. Moreover, the patients with 17p loss without 17q gain do not show difference in prognosis compared to those without 17p loss (*p*-value = 0.49). In contrast, loss of 17p accompanied by loss of 10q reduces survival rates considerably (Fig. 2c). Hence, we conclude that i(17q) and simultaneous loss of 17p and 10q are the risk aberrations in Group 3. After excluding 77 patients with the protective or risk aberrations, the prognosis of the remained 30 patients is separated according to the expression level of 20 core genes (Fig. 2d). The heatmap of core gene expressions of 15 prognostic gene sets shows that protective and risk chromosome aberrations are associated with low and high average expressions of the core genes respectively (Fig. 2e). The expression of the core genes is also significantly associated with subtype in Group 3; the highest expression is observed in subtype of Group 3 γ and histological group of LCA (Additional file 2: Figure S2B).

### Group 4

**Fig. 3:**
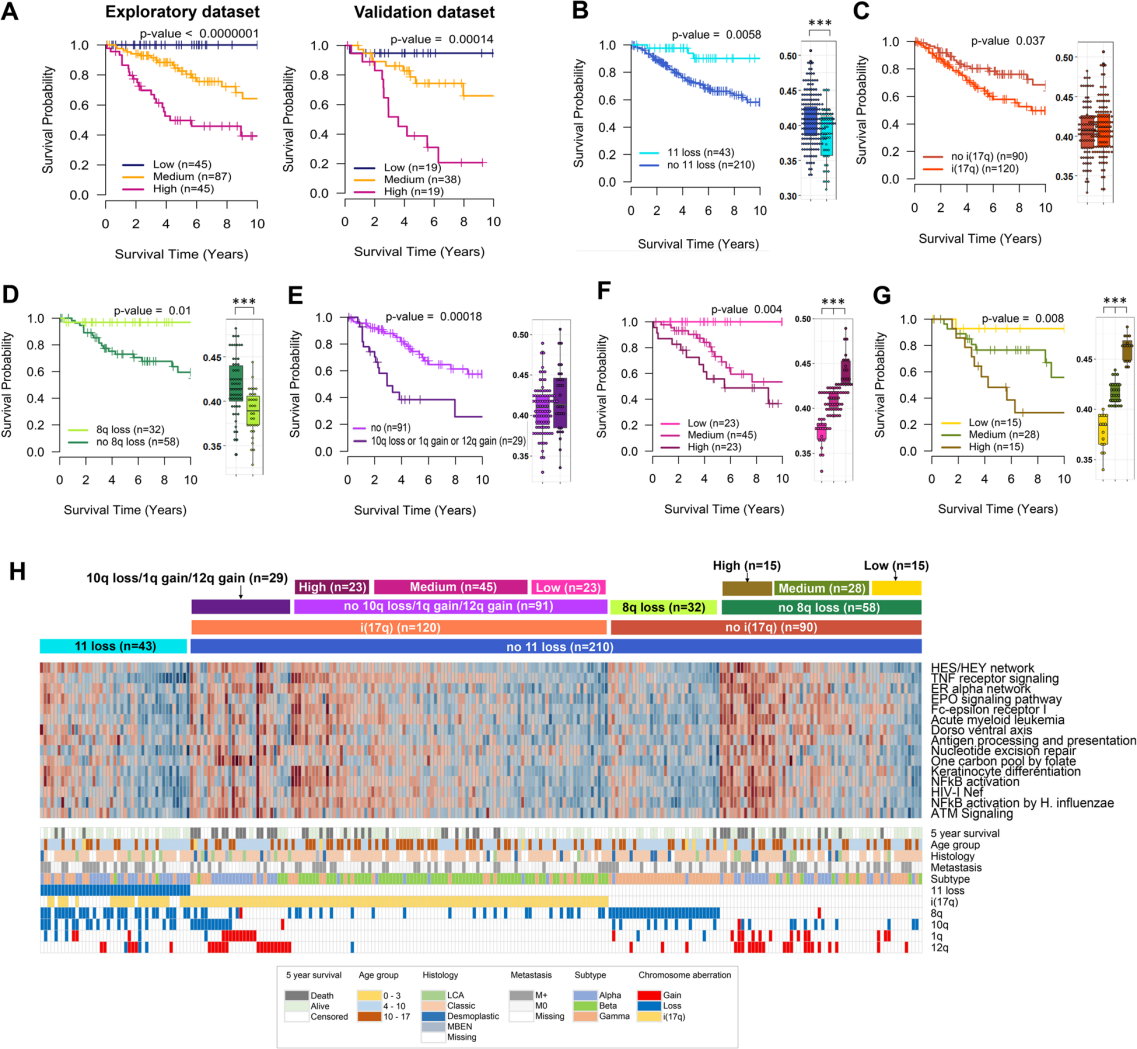
Prognostic power of core genes identified in Group 4. **a**, Kaplan-Meier curves and log-rank tests of the three groups stratified by Q1 (25% quantile) and Q3 (75% quantile) of the average expression of 31 core genes from top two PID pathways. **b**, Kaplan-Meier curve with log-rank test and boxplot of the average expression of 31 core genes of the two groups divided by the status of chr11 loss. **c**, Kaplan-Meier curve with log-rank test and boxplot of the average expression of 31 core genes of the two groups of 210 patients who do not harbor chr11 loss, stratified by the status of i(17q). **d**, Kaplan-Meier curve with log-rank test and boxplot of the average expression of 31 core genes of the two groups of 90 patients who do not harbor chr11 loss and i(17q), stratified by the status of 8q loss. **e**, Kaplan-Meier curve with log-rank test and boxplot of the average expression of 31 core genes of the two groups of 120 patients who harbor i(17q) but not chr11 loss, stratified by the presence of 10q loss, 1q gain, or 12q gain. **f**, Kaplan-Meier curve with log-rank test and boxplot of the average expression of 31 core genes of the three groups of 91 patients who harbor i(17q) but not chr11 loss, 10q loss, 1q gain, and 12q gain, stratified by the average expression of 31 core genes. **g**, Kaplan-Meier curve with log-rank test and boxplot of the average expression of 31 core genes of the three groups of 58 patients who do not harbor chr11 loss, i(17q), and 8q loss, stratified by the average expression of 31 core genes. **h**, Heatmap of expression of core genes from the fifteen prognostic canonical pathways with clinical information, prognostic chromosome aberrations, and subtype [9]. *, Mann–Whitney U test *p*-value < 0.05; **, *p*-value < 0.01; ***, *p*-value < 0.001

In Group 4, total 41 canonical pathways out of 58 canonical pathways significantly detected in GSEA show statistical significance in more than 90% of 1000 bootstrap samples, including “Notch network”, “p53 pathway”, “RhoA signaling pathway”, “NF-κB Signaling Pathway”, “One carbon pool by folate”, “Cell Cycle: G1/S Check Point”, “Integrin-linked kinase signaling”, and several immune response-related canonical pathways such as “TNF receptor signaling pathway”, “Fc-epsilon receptor I signaling in mast cells”, and “Antigen processing and presentation” (Additional file 1: Table S2, Table 3). Stratification of the Group 4 patients using average expression of 31 core genes from the top two PID canonical pathways (“Notch network” and “TNF receptor signaling pathway”) produces clear separation of survival curves in both exploratory and validation datasets (Fig. 3a).

**Table 3:** Top five prognosis-related canonical pathways in Group 3

Database	Name of gene set (GSEA)	Exploratory dataset(n = 177)		Validation dataset (*n* = 76)		1000 bootstrapped datasets (*n* = 253)	Representative core genes**
		HR*	*p*-value	HR*	*p*-value	% of *p*-value < 0.05	
PID	Notch-mediated HES/HEY network	6.49	< 0.0001	3.35	0.009	100.0	HDAC1,NOTCH1,GATA4,HEY1,TCF3,PARP1,RBBP8,E2F1
	TNF receptor signaling pathway	5.31	< 0.0001	5.54	0.000	100.0	FADD,TNFRSF1A,TNFRSF1B,CAV1,IKBKB,GNB2L1,NFKB1
	Validated nuclear estrogen receptor α network	8.13	< 0.0001	5.94	0.006	99.9	MYC,HDAC1,XBP1,TRIM59,CCND1,LCOR,UBE2M
	EPO signaling pathway	3.50	< 0.0001	3.12	0.012	99.9	CBL,CRKL,INPP5D,NFKB1
	Fc-epsilon receptor I signaling in mast cells	4.09	< 0.0001	2.84	0.018	99.9	CBL,GAB2,INPP5D,FCGR2B,IKBKB,NFKB1,FOS
KEGG	Acute myeloid leukemia	4.85	< 0.0001	3.84	0.005	100.0	MYC,AKT3,EIF4EBP1,IKBKB,NFKB1,CCND1
	Dorso ventral axis formation	2.68	0.0004	2.82	0.026	99.5	NOTCH1,ETS2,NOTCH2
	Antigen processing and presentation	1.71	0.0068	2.10	0.004	95.5	B2M,HSPA1A,HLA-F,TAP2,RFXANK,CD74,PDIA3,HLA-DMA
	Nucleotide excision repair	2.22	0.0007	1.40	0.377	93.2	DDB1,POLD4,POLE2,POLE4
	One carbon pool by folate	2.09	< 0.0001	1.13	0.635	92.7	GART,MTHFD2L,MTHFD2,TYMS,DHFR
BIOCARTA	Keratinocyte Differentiation	3.29	0.0001	3.32	0.002	100.0	PRKCD,ETS2,TNFRSF1A,TNFRSF1B,IKBKB,NFKB1,FOS
	NF-kB Signaling Pathway	2.55	0.0001	2.33	0.004	100.0	MYD88,FADD,TNFRSF1A,TNFRSF1B,IKBKB,IL1R1,NFKB1
	HIV-I Nef: negative effector of Fas and TNF	4.71	< 0.0001	3.15	0.011	99.9	PRKCD,FADD,TNFRSF1A,TNFRSF1B,PRKDC,PSEN2, PARP1,NFKB1
	NF-kB activation by Nontypeable *H. influenzae*	5.83	< 0.0001	3.61	0.016	99.8	MYD88,IKBKB,SMAD3,NFKB1
	Cell Cycle: G1/S Check Point	2.64	0.0001	3.54	0.009	99.8	CDC25A,HDAC1,TGFB1,CDKN1A,DHFR,CDK1,SMAD3, CDK2,CCND1,E2F1

* Hazard ratio per 0.1 increment in average relative expression of core genes

** Representative core genes are significantly associated with overall survival (p-value < 0.05) in Cox regression analysis with all samples

Investigation of prognostic chromosome aberration reveals that losses of 11p and 8q are protective aberrations (Additional file 1: Table S5). In previous studies, loss of chr11 has been recognized as an important cytogenetic marker of favorable prognosis in Group 4 [2, 5, 15]. Congruently, we find that 11p loss not accompanied by 11q loss is not a significant prognostic factor (data not shown), and simultaneous loss of 11p and 11q is the most significant prognostic chromosome aberration in Group 4 (Additional file 1: Table S5). The patients with loss of chr11 show excellent survival outcomes and low average expression of 31 prognostic core genes from the top two PID pathways (Fig. 3b). In multivariable Cox regression analysis, the other protective aberration, 8q loss, is no longer significant (*p*-value = 0.23) when the status of chr11 is added to the model. Therefore, we conclude that chr11 loss is the strongest protective chromosome aberration in Group 4. We further investigate prognostic chromosome aberrations after excluding the patients with loss of chr11 in Group 4, and find that i(17q) is a risk chromosome aberration (Additional file 1: Table S6, Fig. 3c). In a similar successive manner, we find that 8q loss is a significant protective aberration in 90 patients after excluding 163 Group 4 patients with chr11 loss or i(17q) (Additional file 1: Table S7, Fig. 3d). Likewise, 10q loss, 1q gain, and 12q gain, are identified as risk aberrations within 120 patients with i(17q) but without loss of chr11 (Additional file 1: Table S8, Fig. 3e). Finally, two groups of remained patients, 91 patients with i(17q) and 58 patients without i(17q), can be further separated into different survival groups respectively according to the average expression of 31 prognostic core genes (Fig. 3f and g), which can be also shown by the heatmap with detailed information (Fig. 3h). On the other hand, high expression level of the 31 core genes is observed in the patients with leptomeningeal metastases (M+) (Additional file 2: Figure S2C).

## Discussion

**Fig. 4:**
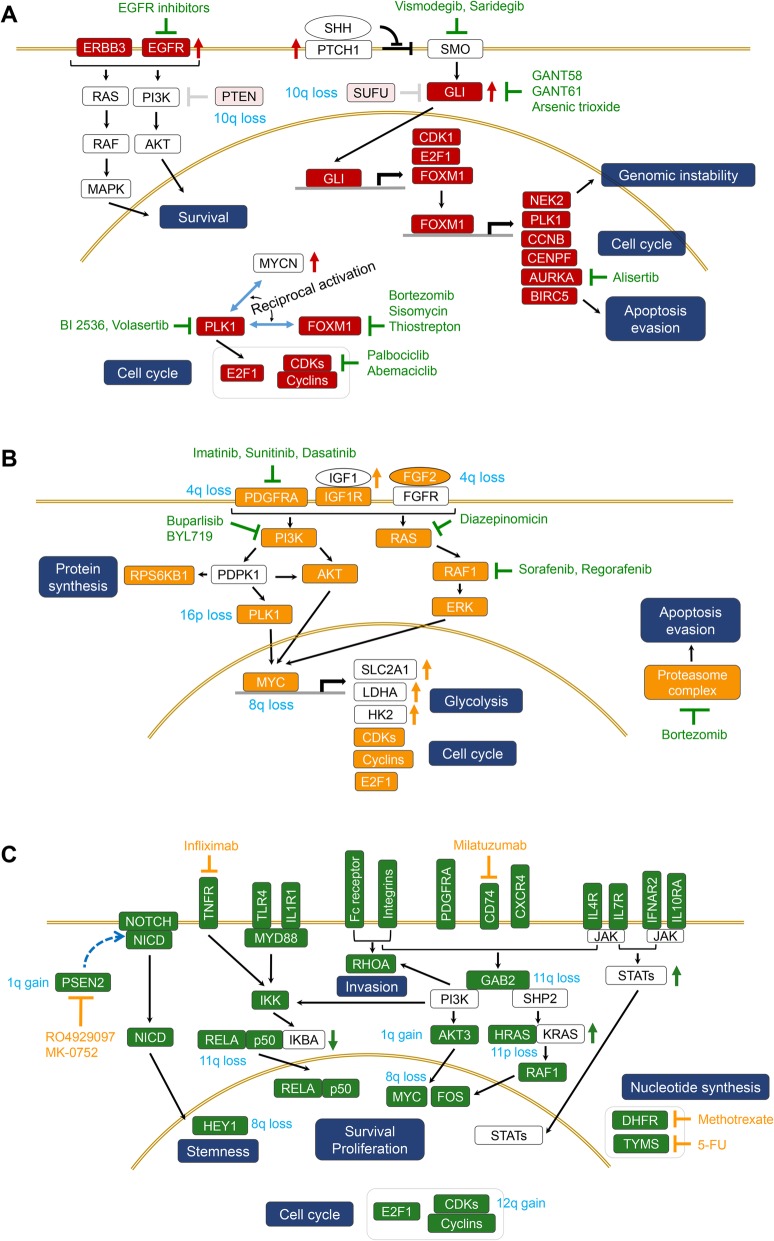
Schematic diagrams of prognosis-related signaling pathways with representative genes and targeted agents. **a**, Prognostic signaling pathways identified in SHH subgroup. **b**, Prognostic signaling pathways identified in Group 3. **c**, Prognostic signaling pathways identified in Group 4. The genes in colored boxes indicate prognosis-related genes detected in SHH (red), Group 3(orange), or Group 4 (green). The genes in the white boxes are linker genes included to connect the signaling pathways. Representative targeted agents in clinical, preclinical or early-phase development are presented. Colored arrow denotes relatively high or low expression of the gene in SHH (red), Group 3(orange), or Group 4 (green) and the detailed boxplot of the gene expression is presented in Additional file 2: Figures S3, S4, or S6. Fc receptors, FCER1G and FCGR2A/B in core gene list of Group 4, Integrins, ITGB1/2 in core gene list of Group 4

**Fig. 5:**
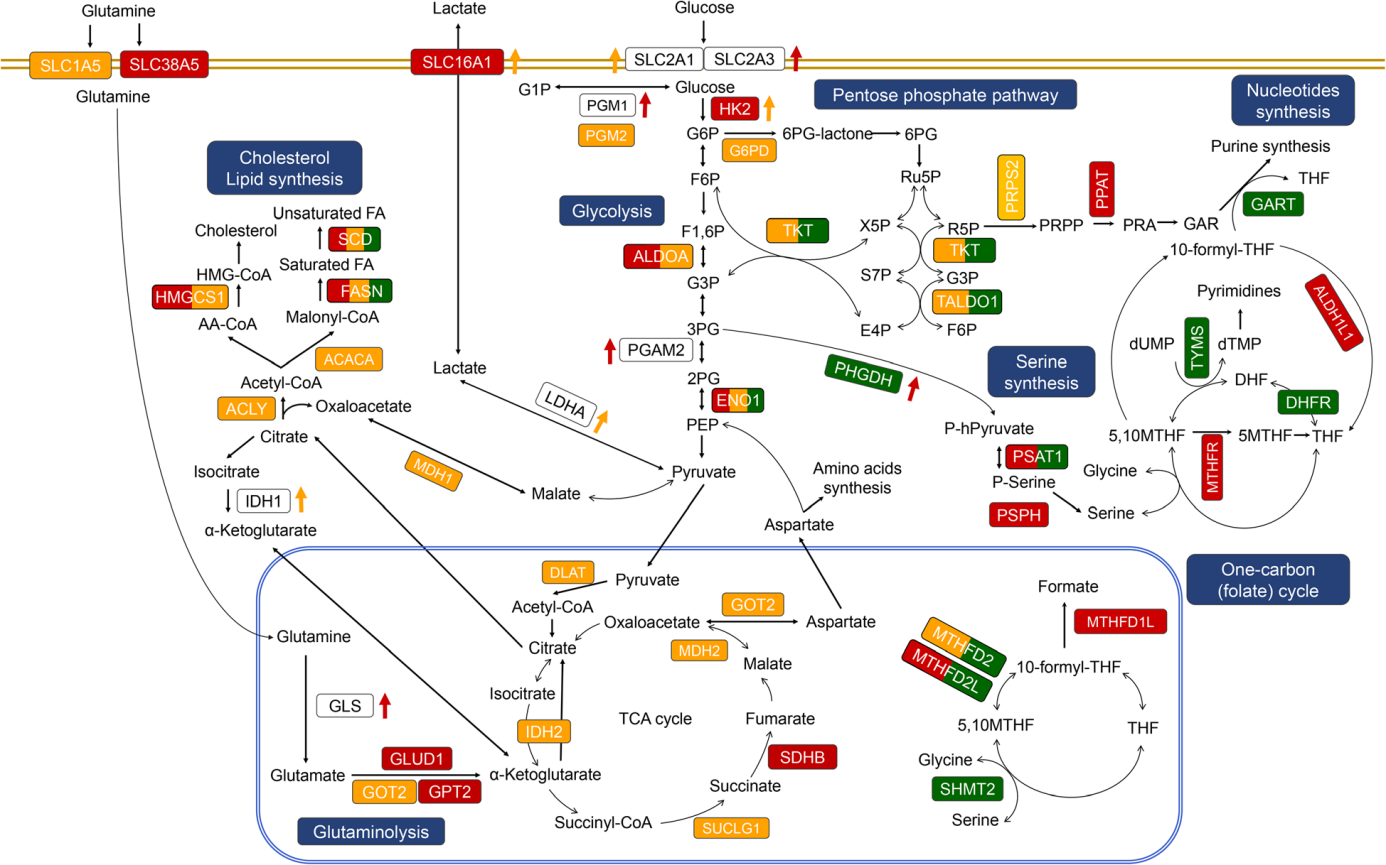
Cancer metabolism-related pathways with prognostic genes identified in medulloblastoma subgroups. The genes in colored boxes indicate prognosis-related genes detected in SHH (red), Group 3(orange), or Group 4 (green). The genes in the white boxes are genes included to complete the metabolic pathways. Colored arrow denotes relatively high or low expression of the gene in SHH (red), Group 3(orange), or Group 4 (green) and the detailed boxplot of the gene expression is presented in Additional file 2: Figures S3, S4, or S6

Current consensus of risk stratification of childhood medulloblastoma has refined the risk stratification according to subgroup and an integration of the clinical markers as well as the molecular, genomic profiles of the tumor [15]. In the SHH subgroup, the risk group was divided by the combination of TP53 mutation, MYCN amplification, and metastasis. For Group 3, the major factors deciding risk were metastasis and MYC amplification. In Group 4, the important segregation points were metastasis and chr11 loss. Despite this huge improvement and modification, ‘unknown’ types of patients have remained, such as Group 3 patients who are non-metastatic but have MYC amplification. Furthermore, metastasis is even more prominent as the major factor in all subgroups, and molecular and genomic mechanisms that are masked by the clinical phenotype of metastasis have remained to be elucidated. More recent studies have examined the presence of subtypes in each medulloblastoma subgroup, characterized by different clinical, genomic, and epigenomic features [9, 10, 16], indicating considerable within-subgroup heterogeneity. Thus, we investigate prognostic signaling pathways in each subgroup of medulloblastoma, to provide molecular basis for characterization of within-subgroup heterogeneity and subgroup-specific therapeutic strategies. Finally, the results are comprehensively summarized in schematic illustrations of prognosis-related signaling pathways (Fig. 4) and metabolic pathways (Fig. 5).

### SHH subgroup

In SHH subgroup, majority of the prognostic gene sets are related to cell cycle and mitosis such as the canonical pathways of “p53 pathway”, “PLK1 signaling events”, “FOXM1 transcription factor network”, “Aurora B signaling”, and “Cyclins/CDKs”. In SHH signaling pathway, FOXM1 has been known to be a downstream target gene of GLI transcription factor that is activated by SMO relieved by hedgehog ligands (Fig. 4a) [17–19]. Consistently, we observe a considerable correlation between the expressions of GLI2 and FOXM1 (Pearson’s correlation coefficient = 0.48, *p*-value = 2.9 × 10^− 8^). Additionally, as expected, the expressions of GLI1 and GLI2 are profoundly high in SHH subgroup (Additional file 2: Figure S3A) and high expression level of GLI2 is significantly associated with prognosis (HR = 1.45 per 0.1 increment of relative expression, p-value = 0.0074). As a transcription factor, FOXM1 subsequently activates essential genes for mitotic progression including PLK1 [20–22]. FOXM1 and PLK1 are cooperatively overexpressed in various cancers [23–25] and identified as potential therapeutic targets [22, 26–28]. They play crucial roles in mitosis entry by complicated inter-activating relationship [29]. Such a reciprocal activation is also reported between PLK1 and MYCN [30], one of the well-known genes highly expressed in SHH medulloblastomas. Besides the core genes detected in GSEA, two genes encoding ErbB family proteins, EGFR and ERBB3, are observed to be prognostic in gene-wise univariable Cox regressions (HR = 1.35 per 0.1 increment of relative expression and *p*-value = 0.045 for EGFR; HR = 1.55 per 0.1 increment of relative expression and *p*-value < 0.00001 for ERBB3). The expression of EGFR is relatively high in SHH subgroup. In contrast, only a small subset of tumors shows a very high level of ERBB3 expression (Additional file 2: Figure S3A).

Gain of 9p, frequently co-occurred with loss of 9q, was recognized in SHH tumors [31], however, its association with prognosis has not been reported. In our result, favorable prognosis is observed in the SHH patients with gain of 9p. It has been well known that 9p contains two important tumor suppressor genes, CDKN2A and CDKN2B. Indeed, median expression of CDKN2B was slightly higher in SHH patients with 9p gain with statistical significance (Additional file 2: Figure S3B). We also detect that the SHH patients with i(17q) or simultaneous loss of 10q, 17p, or 14q have the worst survival outcome (median survival time = 1.59 years) (Fig. 1c). As expected, the expressions of important tumor suppressors, TP53 and PTEN/SUFU, are significantly or marginally significantly reduced with the corresponding chromosome aberrations, 17p loss and 10q loss in SHH medulloblastomas (Additional file 2: Figure S3B). In Fig. 4a, several anti-cancer agents targeting deregulated signaling pathways in SHH subgroup are presented. Proteasome inhibitors (bortezomib) and thiazole antibiotics (sisomicin A and thiostrepton) target FOXM1 and induce apoptosis [32, 33]. The mitotic kinases PLK1 and AURKA/B are blocked by the small-molecule selective inhibitors BI 2536/volasertib and alisertib, respectively [34, 35]. Palbociclib and abemaciclib are selective CDK4/6 inhibitors [36]. SHH signaling is also blocked by the SMO inhibitors vismodegib and saridegib and the GLI inhibitors GANT58, GANT61, and arsenic trioxide. Up-regulated EGFR is blocked by various inhibitors including gefitinib, erlotinib, and cetuximab.

### Group 3

In Group 3, two receptor tyrosine kinases (PDGFRA and IGF1R), FGF2 also known as basic fibroblast growth factor (bFGF), and the genes in their downstream signaling pathways such as PI3K/AKT and MAPK/ERK are detected as prognostic core genes (Fig. 5a). Furthermore, IGF1 is observed to be most highly expressed in Group 3 (Additional file 2: Figure S4A). High expression of MYC is confirmed in Group 3 tumors, and its target genes involved in glycolysis including SLC2A1 (GLUT1), LDHA, and HK2 are noticed to be highly expressed as well (Additional file 2: Figure S4A). Additionally, many genes encoding proteasomes, cyclins, and CDKs are also detected as prognostic genes.

The most favorable survival rate is observed in the Group 3 patients who have simultaneous loss of 4q, 8q, or 16p, which can be possibly explained by the loss of the oncogenic core genes such as MYC on 8q, FGF2 and PDGFRA on 4q, and PLK1 on 16p. Indeed, the expressions of the four genes are significantly or marginally significantly low in accordance with the corresponding chromosomal deletions (Additional file 2: Figure S4B). On the other hand, i(17q) and simultaneous loss of 17p and 10q are associated with poor survival outcome in Group 3, which is also observed in SHH subgroup.

Anti-cancer agents targeting PDGFRA include receptor tyrosine kinase (RTK) inhibitors such as imatinib, sunitinib, and dasatinib. Downstream of RTKs is targeted by PI3K inhibitors (buparlisib and BYL719) [37, 38] and Ras-Raf-MEK-ERK pathway inhibitors (diazepinomicin, sorafenib and regorafenib) [39–41]. Up-regulated proteasome complex is blocked by bortezomib.

### Group 4

In Group 4, “Notch network” genes such as NOTCH1, NOTCH2, and HEY1 are included in the prognostic core genes (Additional file 1: Table S2, Table 3). In addition, high expressions of many genes encoding immune receptors are also associated with poor survival outcome, including cytokine receptors (TNFRSF1A/B, ITGB1/2, IFNAR2, IL1R1, IL4R, IL7R, IL10RA, CXCR4, and CD74), Toll-like receptors (TLR3/4/5), and Fc receptors (FCER1G and FCGR2A/B). Interestingly, the expressions of NOTCH1, NOTCH2 or NOTCH3 are highly correlated with those of many prognostic immune receptors (Additional file 2: Figure S5), implying that Notch signaling might be important in the regulation of tumor immune response or tumor microenvironment, which, in fact, has been reported in many studies [42, 43]. For example, activation of Notch signaling induced recruitment of tumor-associated macrophage (TAM) [44] and anti-Notch treatment reduced immune-suppressive M2 TAM infiltration [45] or inhibited tumor-induced T-cell tolerance mediated by Myeloid-derived suppressor cells [42], thus underscoring possible immunotherapeutic opportunities in Group 4 medulloblastoma patients with relative activation of Notch signaling. Notch signaling is also known to be closely related to stemness of medulloblastoma cell and essential for maintenance of stemness in brain tumor initiating cell [46, 47]. Enhanced expressions of the genes involved in nucleotide synthesis (DHFR and TYMS), cell cycle, and NF-κB, PI3K/AKT, or RHOA pathways are also associated with poor prognosis. Several genes in JAK-STAT pathway, the major downstream signaling of cytokine receptors, are most highly expressed in Group 4, including JAK2, STAT2, STAT3, STAT5A, and STAT5B (Additional file 2: Figure S6A). Besides, “Validated nuclear estrogen receptor α network” is recognized as the deregulated signaling pathway in Group 4 (Table 3). Congruently, expression of estrogen-related receptor γ (ESRRG) is found to be considerably high in Group 4 tumors (Additional file 2: Figure S6A).

The Group 4 patients with loss of chr11 or loss of 8q without i(17q) show excellent survival rates (Fig. 3b and d), which could possibly be explained by reduced expressions of prognostic core genes such as HRAS on 11p, GAB2 and RELA on 11q, and MYC and HEY1 on 8q (Additional file 2: Figure S6B). Likewise, the poor survival outcome of the Group 4 patients with loss of 10q, or gains of 1q or 12q accompanied by i(17q) might be related to reduced expression of PTEN on 10q, or elevated expressions of AKT3 and PSEN2 on 1q and CDK2 on 12q (Additional file 2: Figure S6B, Fig. 4c).

The Notch signaling pathway is blocked by MK-0752 and RO4929097, inhibitors of γ-secretase that mediates the cleavage of the Notch intracellular domain (NICD), which is translocated to the nucleus and activates the transcription of NOTCH target genes [48, 49]. Considering the prognostic significance of immune receptors, NF-κB signaling, and JAK-STAT pathway, anti-inflammatory agents and monoclonal antibodies against immune receptors (milatuzumab and infliximab) may provide therapeutic interventions in Group 4 [50–52]. Anti-cancer agents targeting nucleotide biosynthesis pathways include a DHFR inhibitor (methotrexate) and a TYMS inhibitor (5-FU).

### Prognostic metabolic pathways in medulloblastoma subgroups

Finally, we also detect several metabolic pathways that are known to be reprogrammed in cancer as prognostic canonical pathways such as “Alanine, aspartate, and glutamate metabolism” in SHH subgroup, “Pentose phosphate pathway” and “TCA cycle” in Group 3, and “One carbon pool by folate” in Group 4. Accordingly, focusing on the widely known metabolic pathways modified in cancer cells [53–57], a comprehensive schematic diagram is presented in Fig. 5 based on not only the prognostic core genes obtained in GSEA but also the genes significantly detected in gene-wise univariable Cox regression analysis (*p*-value < 0.05) or highly expressed in a particular medulloblastoma subgroup. Several genes are commonly detected as prognostic genes in all three subgroups, SHH subgroup, Group 3, and Group 4, including ENO1 encoding enolase 1 in glycolytic pathway and FASN and SCD encoding fatty acid synthase and stearoyl-CoA desaturase respectively in fatty acid synthesis pathway. Many genes involved in glycolysis or glutaminolysis are prognostic or highly expressed in SHH subgroup or Group 3. The genes involved in one carbon (folate) cycle or serine synthesis are prognostic mainly in SHH subgroup or Group 4. Two important genes in pentose phosphate pathway, TKT and TALDO1, are associated with poor prognosis in Group 3 and Group 4. It has long been recognized that activation of those metabolic pathways supports tumor cell anabolism for rapid proliferation and plays a crucial role in maintenance of energy and redox homeostasis in cancer cells [53–58]. Thus, targeting the key enzymes in the altered tumor metabolic pathways is now being actively investigated as a new potential chemotherapeutic strategy [59, 60], which also shed light on treatment for medulloblastoma.

## Conclusions

Data from a decade of genomic analysis have completely changed the definition and diagnosis of medulloblastoma. Research is now progressing toward the identification of prognostic and therapeutic applications of this accumulated genomic data. Our primary and ultimate goal is to identify the genes or signaling pathways that are essentially responsible for the prognosis of each established medulloblastoma subgroup. The prognostic genes detected in a particular subgroup are not necessarily highly expressed in the given subgroup compared to other subgroups. Rather, we find that most of the subgroup-specific prognosis-related genes show almost the same levels or even lower levels of expression, indicating complicated prognostic molecular determinants. Thus, our results suggest that we need to consider not only the genes that are highly expressed or amplified but also those with average or relatively low level of expression to expand the therapeutic targets and strategies.

## Additional files

Additional file 1: **Table S1**. Goodness-of-fit tests for Cox proportional hazards model. **Table S2**. Subgroup-specific enriched gene sets (canonical pathways) and core genes positively and negatively associated with poor prognosis. **Table S3**. Cox regression analysis with chromosome aberrations occurred in more than 10% of patients in SHH subgroup. **Table S4**. Cox regression analysis with chromosome aberrations occurred in more than 10% of patients in Group 3 **Table S5**. Cox regression analysis with chromosome aberrations occurred in more than 10% of patients in Group4. **Table S6**. Cox regression analysis with chromosome aberrations in Group4 after excluding the patients with chr11 loss. **Table S7**. Cox regression analysis with chromosome aberrations in Group4 patients without chr11 loss nor i(17q). **Table S8**. Cox regression analysis with chromosome aberrations in Group4 patients harboring i(17q) but not chr11 loss. (XLSX 1264 kb)

Additional file 2: **Figure S1.** Kaplan-Meier subgroup analysis. A, 10-year Kaplan-Meier subgroup analysis in the exploratory dataset. B, 10-year Kaplan-Meier subgroup analysis in the validation dataset. **Figure S2.** Association between the average relative expression of prognostic core genes and subtype and clinical characteristics. A, Boxplots of relative expression of 31 core genes from the top two PID pathways in SHH subgroup. B, Boxplots of relative expression of 20 core genes from the top two PID pathways in Group 3. C, Boxplots of relative expression of 31 core genes from the top two PID pathways in Group 4. A, large cell/anaplastic (LCA); C, Classic; D, Desmoplastic; M, Medulloblastoma with extensive nodularity (MBEN). *, Mann–Whitney U test *p*-value < 0.05; **, *p*-value < 0.01; ***, *p*-value < 0.001. **Figure S3.** Differential expressions of the prognostic genes identified in SHH subgroup. A, Boxplots of gene expressions in four subgroups. B, Differential gene expressions with Mann–Whitney U test *p*-values according to the status of chromosome aberrations in SHH subgroup. **Figure S4.** Differential expressions of the prognostic genes identified in Group 3. A, Boxplots of gene expressions in four subgroups. B, Differential gene expressions with Mann–Whitney U test *p*-values according to the status of chromosome aberrations in Group 3. **Figure S5.** Correlation between expressions of NOTCH and those of immune receptors. Scatter plots with regression lines are presented with Pearson’s correlation coefficients (r) calculated between the expressions of NOTCH1 (A), NOTCH2 (B), or NOTCH3 (C) and those of immune receptors that show r > 0.4. **Figure S6.** Differential expressions of the prognostic genes identified in Group 4. A, Boxplots of gene expressions in four subgroups. B, Differential gene expressions with Mann–Whitney U test p-values according to the status of chromosome aberrations in Group 4. (PDF 1629 kb)

## Data Availability

The datasets analyzed during the current study are available in the Gene Expression Omnibus (GSE85217).

## Abbreviations

ACACA: Acetyl-CoA Carboxylase Alpha
ACLY: ATP Citrate Lyase
AKT: AKT Serine/Threonine Kinase
ALDOA: Aldolase Fructose-Bisphosphate A
AURKA: Aurora kinase A
bFGF: basic fibroblast growth factor
BIOCARTA: BioCarta
BIRC5: Baculoviral IAP Repeat Containing 5 (Survivin)
CD74: CD74 Molecule
CDK: Cyclin-dependent kinase
CENPF: Centromere protein F
CXCR4: C-X-C Motif Chemokine Receptor 4
DHFR: dihydrofolate reductase
DLAT: Dihydrolipoamide S-Acetyltransferase
E2F: E2F Transcription Factor
EGFR: Epidermal Growth Factor Receptor
ENO1: Enolase 1
ERBB3: Erb-B2 Receptor Tyrosine Kinase 3
ERK: Extracellular signal–regulated kinase
FASN: Fatty Acid Synthase
FGF2: Fibroblast growth factor 2
FGFR: Fibroblast growth factor receptor
FOS: Fos proto-oncogene AP-1 transcription factor subunit
FOXM1: Forkhead box protein M1
G6PD: Glucose-6-Phosphate Dehydrogenase
GAB2: GRB2 Associated Binding Protein 2
GART: phosphoribosylglycinamide formyltransferase phosphoribosylglycinamide synthetase phosphoribosylaminoimidazole synthetase
GLI: GLI Family Zinc Finger
GLS: Glutaminase
GLUD1: Glutamate Dehydrogenase 1
GOT2: Glutamic-Oxaloacetic Transaminase 2
GPT2: Glutamic--Pyruvic Transaminase 2
GSEA: Gene Set Enrichment Analysis
HEY1: Hes Related Family BHLH Transcription Factor With YRPW Motif 1
HK2: Hexokinase 2
HMGCS1: 3-Hydroxy-3-Methylglutaryl-CoA Synthase 1
HR: hazard ratio
HRAS: HRas Proto-Oncogene GTPase
i(17q): isochromosome 17q
IDH1: Isocitrate Dehydrogenase (NADP(+)) 1 Cytosolic
IDH2: Isocitrate Dehydrogenase (NADP(+)) 2 Mitochondrial
IFNAR2: Interferon Alpha And Beta Receptor Subunit 2
IGF1: Insulin Like Growth Factor 1
IGF1R: Insulin Like Growth Factor 1 Receptor
IKBA: NFKB Inhibitor Alpha (NFKBIA)
IKK: IκB kinase
IL1R1: Interleukin 1 Receptor Type 1
IL4R: Interleukin 4 Receptor
IL7R: Interleukin 7 Receptor
JAK: Janus Kinase
KEGG: Kyoto Encyclopedia of Genes and Genomes
KRAS: KRAS Proto-Oncogene GTPase
LCA: Large cell/anaplastic
LDH1L1: Aldehyde Dehydrogenase 1 Family Member L1
LDHA: Lactate Dehydrogenase A
MAGIC: Medulloblastoma Advanced Genomic International Consortium
MAPK: Mitogen-Activated Protein Kinase
MDH1: Malate Dehydrogenase 1
MDH2: Malate Dehydrogenase 2
MTHFD1L: Methylenetetrahydrofolate Dehydrogenase (NADP+ Dependent) 1 Like
MTHFD2: Methylenetetrahydrofolate Dehydrogenase (NADP+ Dependent) 2 Methenyltetrahydrofolate Cyclohydrolase
MTHFD2L: Methylenetetrahydrofolate Dehydrogenase (NADP+ Dependent) 2 Like
MTHFR: Methylenetetrahydrofolate Reductase
MYC: MYC Proto-Oncogene BHLH Transcription Factor
MYCN: MYCN Proto-Oncogene BHLH Transcription Factor
NEK2: NIMA Related Kinase 2
NICD: Notch intracellular domain
p50: Nuclear Factor Kappa B Subunit 1 (NFKB1)
PDGFRA: Platelet Derived Growth Factor Receptor Alpha
PDPK1: 3-Phosphoinositide Dependent Protein Kinase 1
PGAM2: Phosphoglycerate Mutase 2
PGM1: Phosphoglucomutase 1
PGM2: Phosphoglucomutase 2
PHGDH: Phosphoglycerate Dehydrogenase
PI3K: Phosphoinositide 3-kinase
PI3K/Akt: Class I PI3K signaling events mediated by Akt
PID: Pathway Interaction Database
PLK1: Polo-like kinase 1
PPAT: Phosphoribosyl Pyrophosphate Amidotransferase
PRPS2: Phosphoribosyl Pyrophosphate Synthetase 2
PSAT1: Phosphoserine Aminotransferase 1
PSEN2: Presenilin 2
PSPH: Phosphoserine Phosphatase
PTCH1: Patched 1
RAF: RAF kinases
RAF1: Raf-1 Proto-Oncogene Serine/Threonine Kinase
Ras/Rho on G1 to S: Influence of Ras and Rho proteins on G1 to S Transition
RELA: RELA Proto-Oncogene NF-KB Subunit
RHOA: Ras Homolog Family Member A
RPS6KB1: Ribosomal Protein S6 Kinase B1 (p70S6K)
SCD: Stearoyl-CoA Desaturase
SDHB: Succinate Dehydrogenase Complex Iron Sulfur Subunit B
SHH: Sonic hedgehog
SHMT2: Serine Hydroxymethyltransferase 2
SLC16A1: Solute Carrier Family 16 Member 1
SLC1A5: Solute Carrier Family 1 Member 5
SLC2A1: Solute Carrier Family 2 Member 1 (GLUT1)
SLC2A3: Solute Carrier Family 2 Member 3 (GLUT3)
SLC38A5: Solute Carrier Family 38 Member 5
SMO: Smoothened Frizzled Class Receptor
STAT: signal transducer and activator of transcription
SUCLG1: Succinate-CoA Ligase Alpha Subunit
SUFU: SUFU Negative Regulator of Hedgehog Signaling
TALDO1: Transaldolase 1
TAM: Tumor-associated macrophage
TKT: Transketolase
TLR4: Toll-like receptor 4
TNFR: tumor necrosis factor receptor
TYMS: Thymidylate Synthetase
