# Supplementary figures and images for: Subgroup-specific prognostic signaling and metabolic pathways in pediatric medulloblastoma
Source: BMC Cancer. 2019 Jun 11;19:571. doi: 10.1186/s12885-019-5742-x (PMC6560914; doi:10.1186/s12885-019-5742-x)

Figure S1

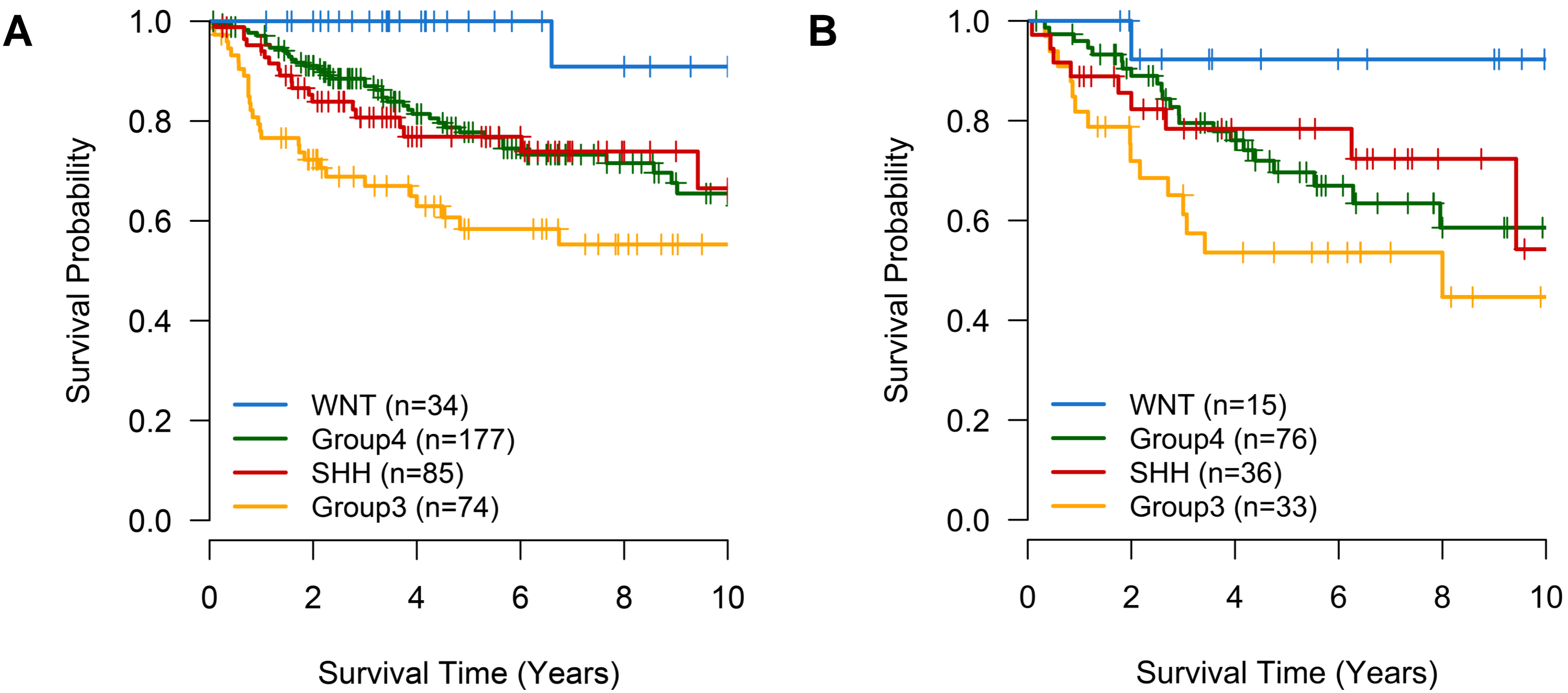

Figure S2

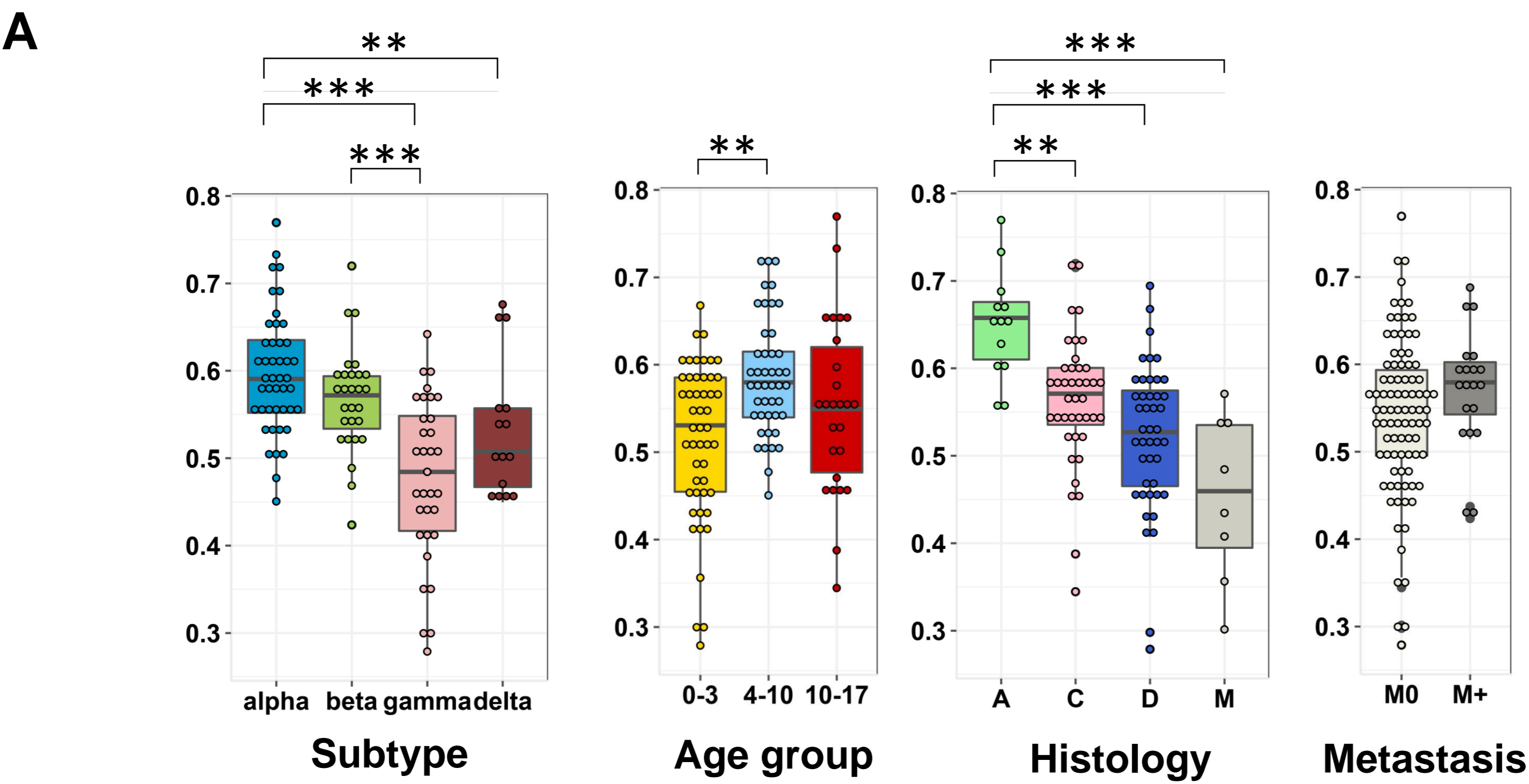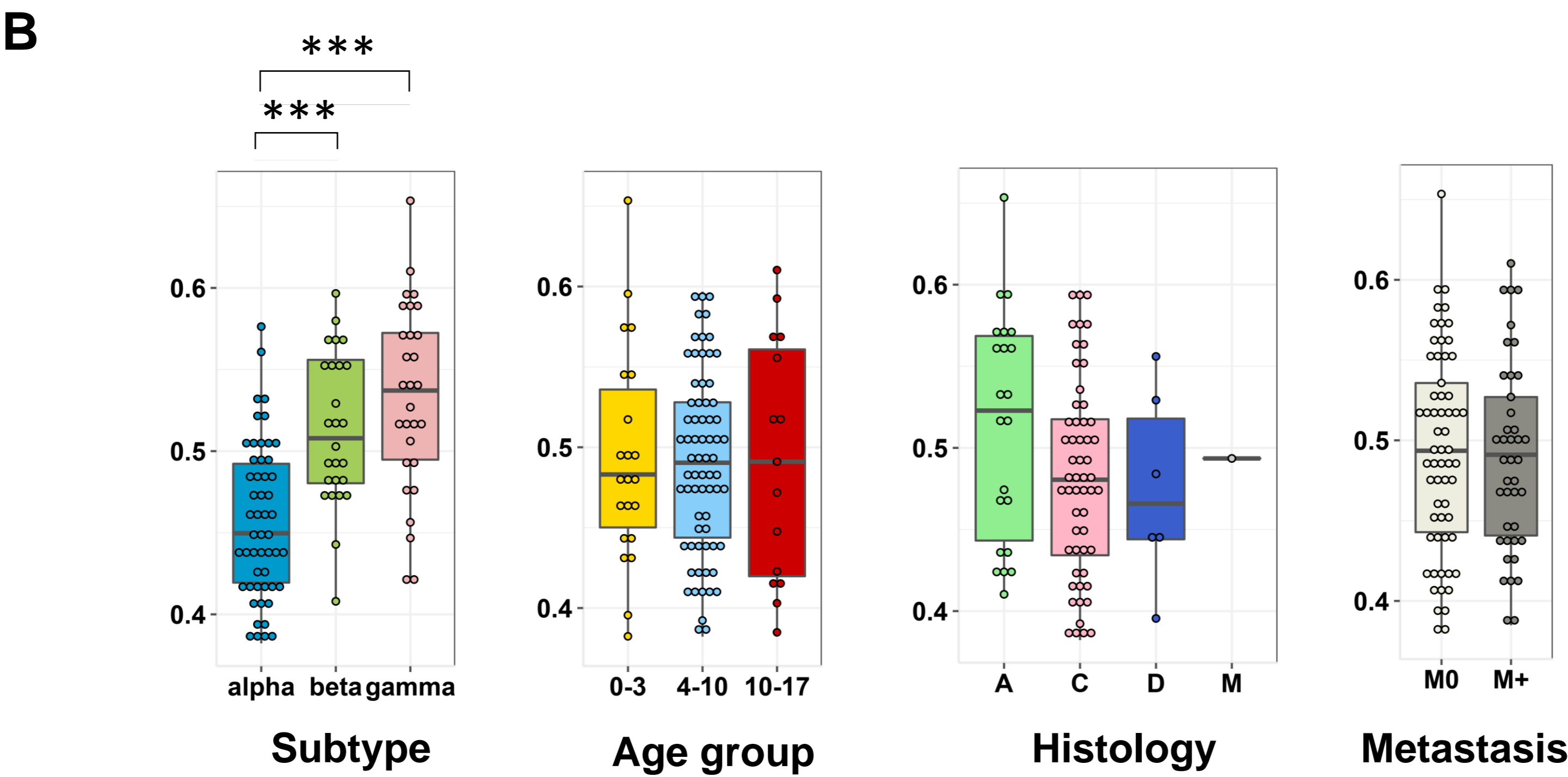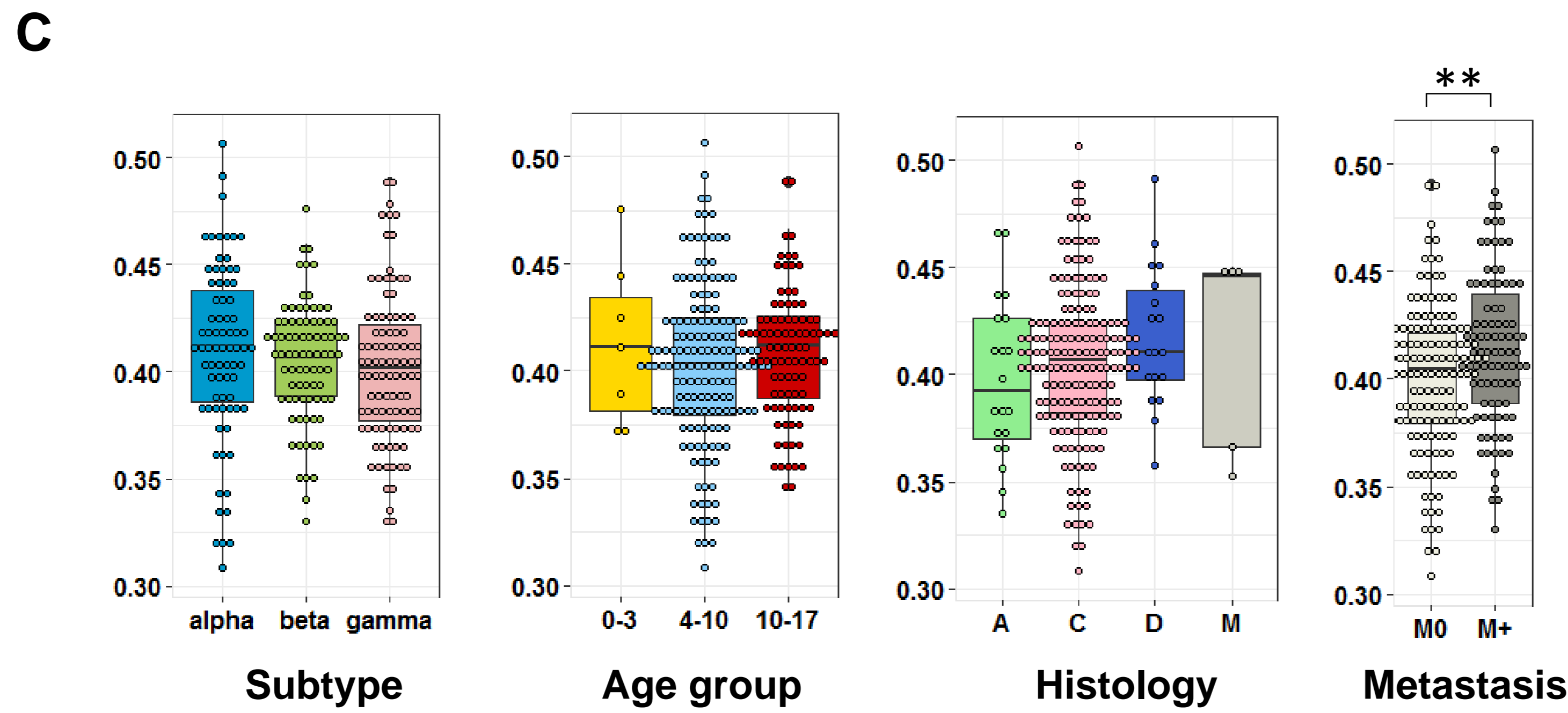

Figure S3

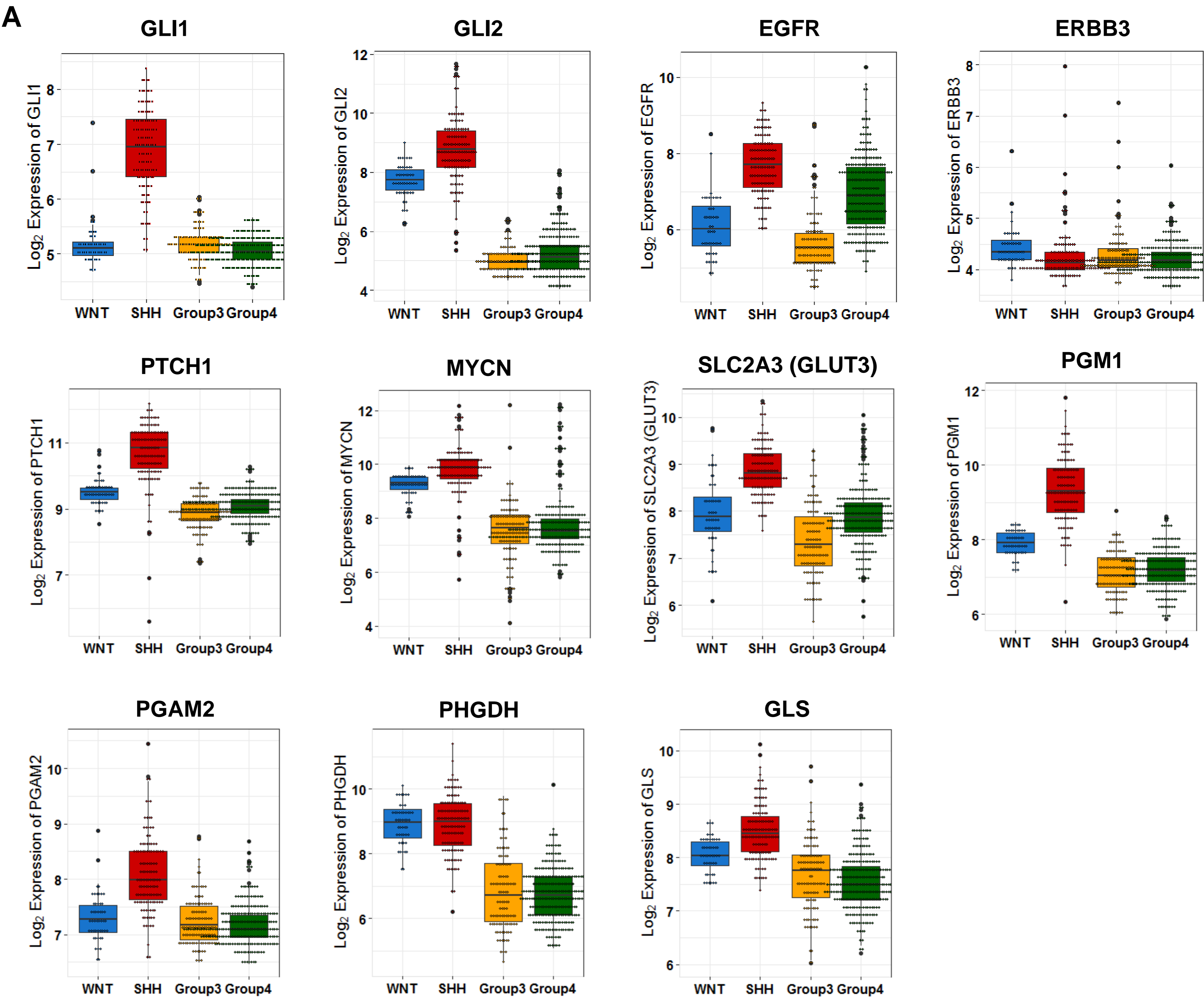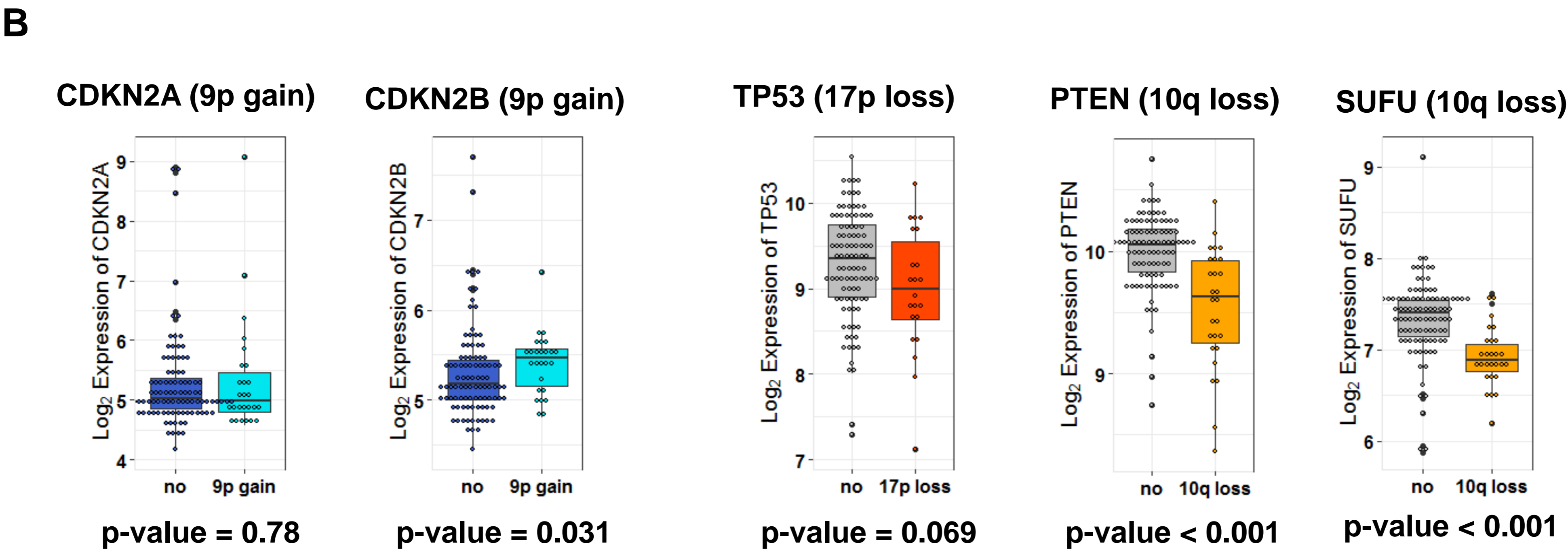

Figure S4

A

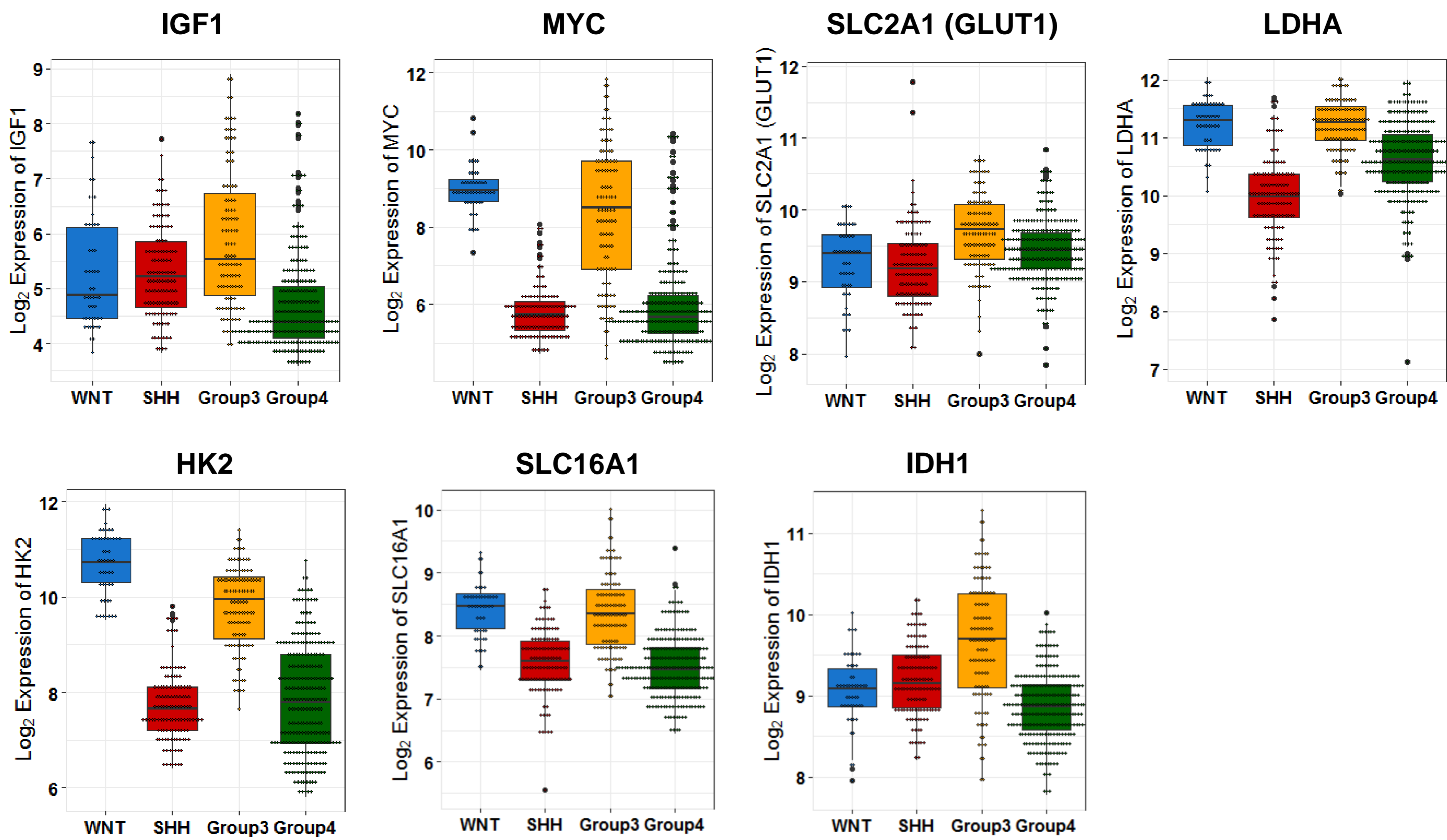

B

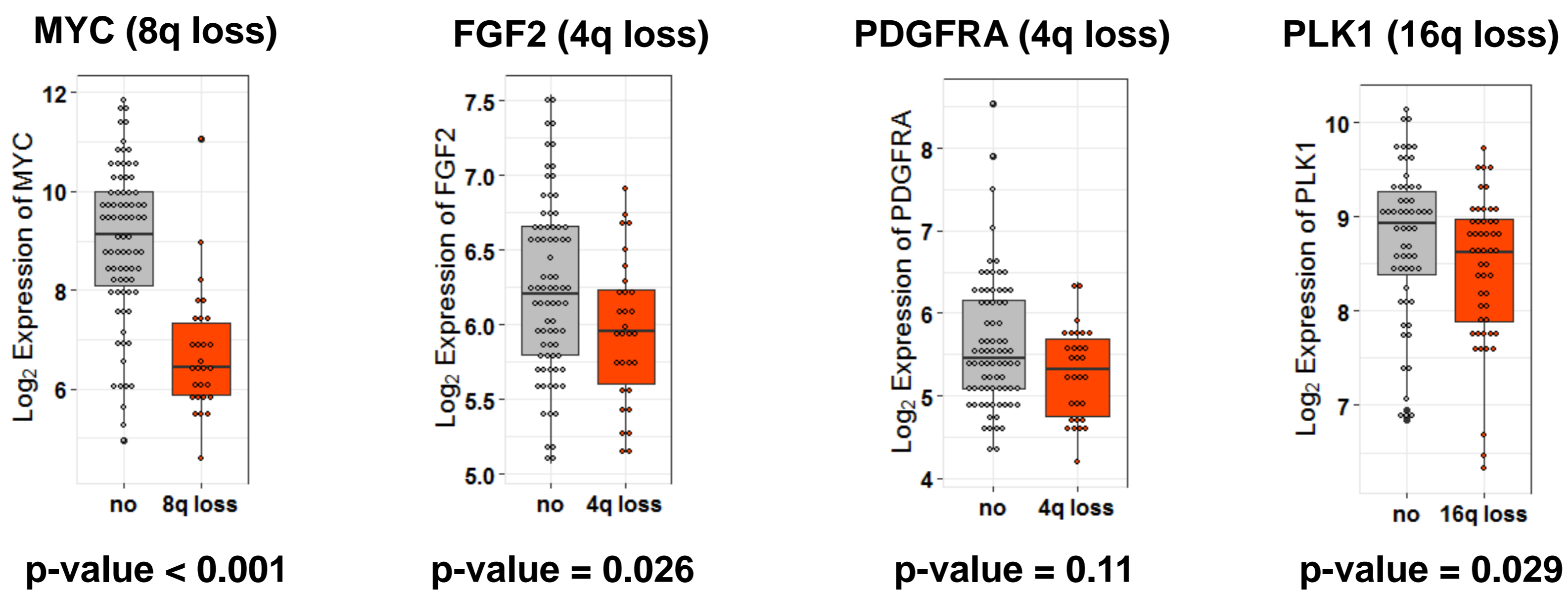

Figure S5

A

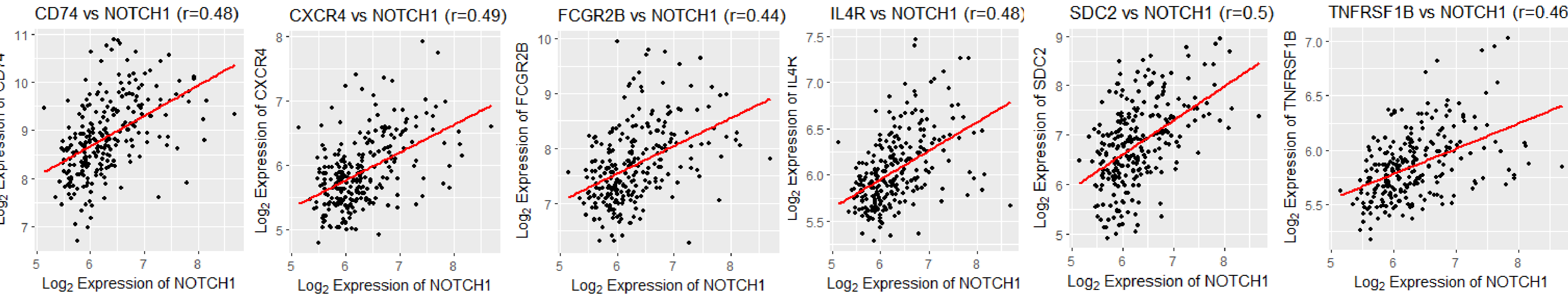

B

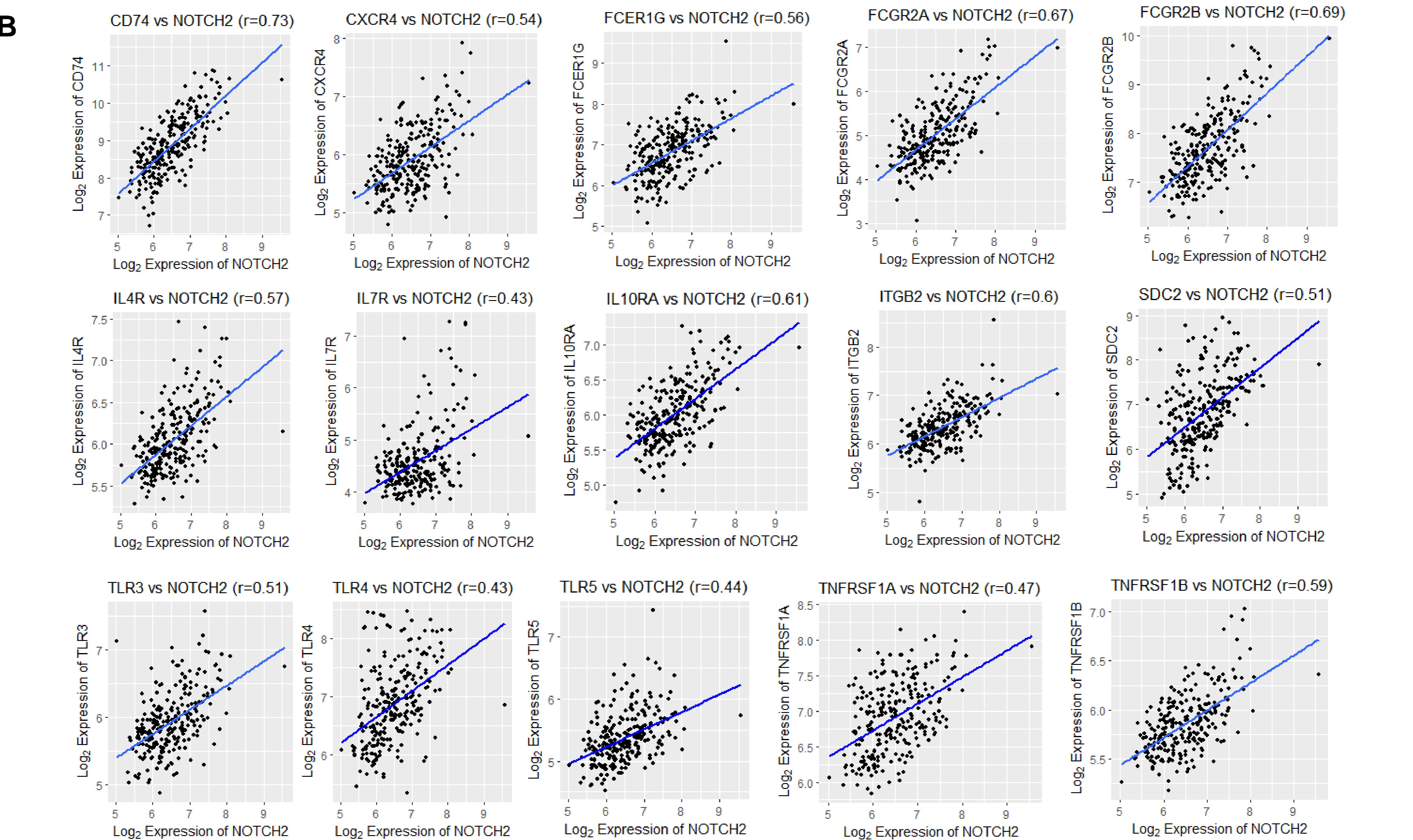

C

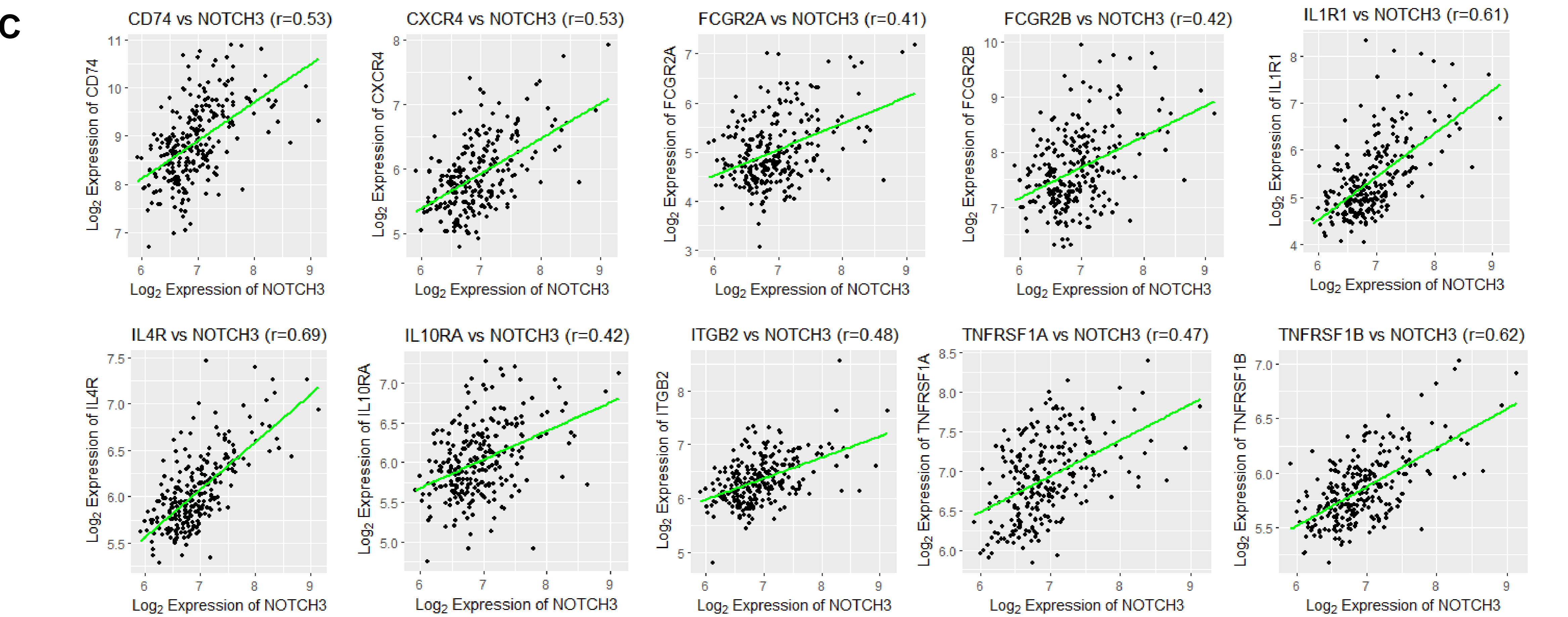

Figure S6

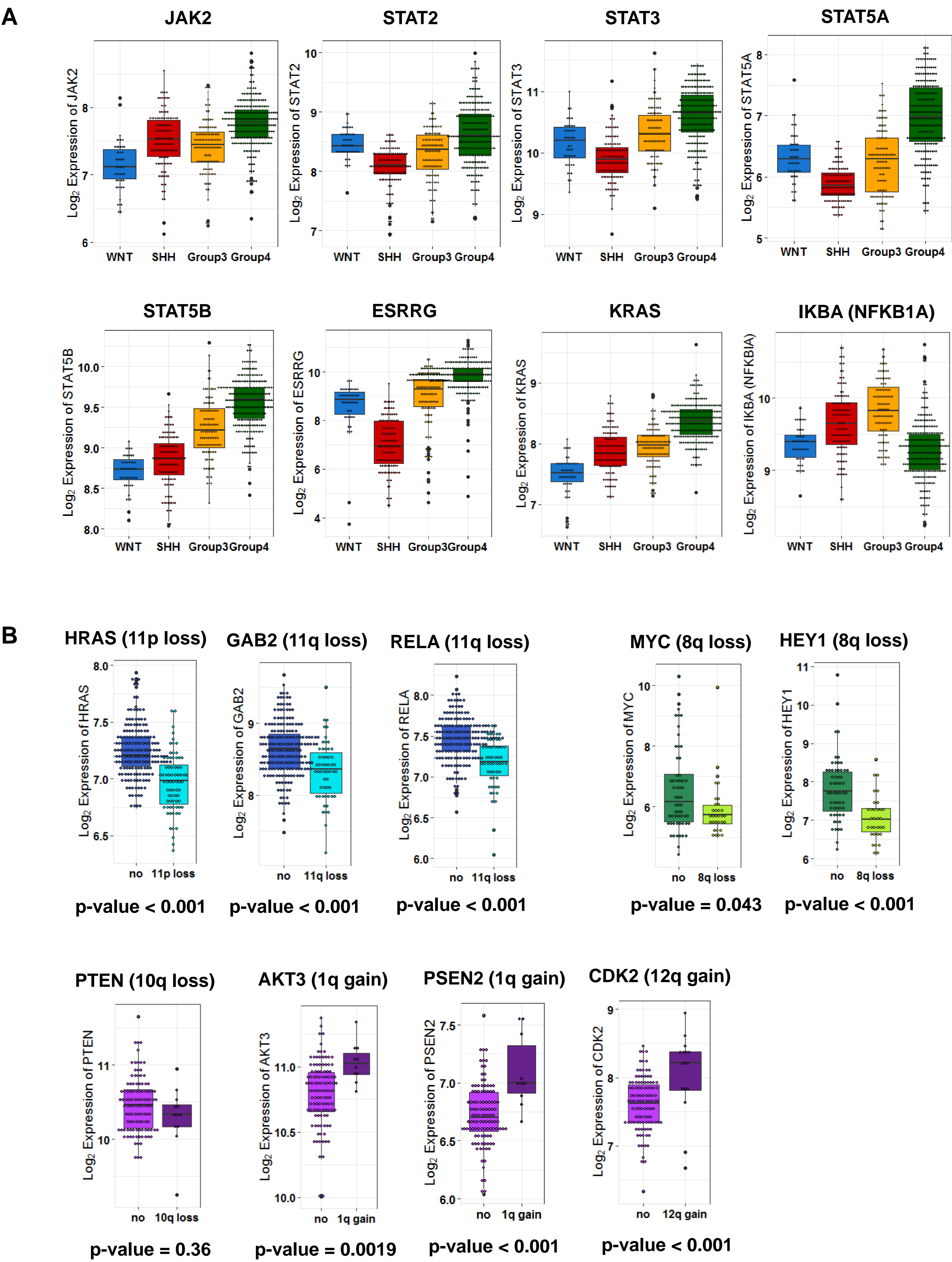

Supplement: Supplementary file 2 — Figure S1. Kaplan-Meier subgroup analysis. A, 10-year Kaplan-Meier subgroup analysis in the exploratory dataset. B, 10-year Kaplan-Meier subgroup analysis in the validation dataset. Figure S2. Association between the average relative expression of prognostic core genes and subtype and clinical characteristics. A, Boxplots of relative expression of 31 core genes from the top two PID pathways in SHH subgroup. B, Boxplots of relative expression of 20 core genes from the top two PID pathways in Group 3. C, Boxplots of relative expression of 31 core genes from the top two PID pathways in Group 4. A, large cell/anaplastic (LCA); C, Classic; D, Desmoplastic; M, Medulloblastoma with extensive nodularity (MBEN). *, Mann–Whitney U test p-value < 0.05; **, p-value < 0.01; ***, p-value < 0.001. Figure S3. Differential expressions of the prognostic genes identified in SHH subgroup. A, Boxplots of gene expressions in four subgroups. B, Differential gene expressions with Mann–Whitney U test p-values according to the status of chromosome aberrations in SHH subgroup. Figure S4. Differential expressions of the prognostic genes identified in Group 3. A, Boxplots of gene expressions in four subgroups. B, Differential gene expressions with Mann–Whitney U test p-values according to the status of chromosome aberrations in Group 3. Figure S5. Correlation between expressions of NOTCH and those of immune receptors. Scatter plots with regression lines are presented with Pearson’s correlation coefficients (r) calculated between the expressions of NOTCH1 (A), NOTCH2 (B), or NOTCH3 (C) and those of immune receptors that show r > 0.4. Figure S6. Differential expressions of the prognostic genes identified in Group 4. A, Boxplots of gene expressions in four subgroups. B, Differential gene expressions with Mann–Whitney U test p-values according to the status of chromosome aberrations in Group 4. (PDF 1629 kb) [file 12885_2019_5742_MOESM2_ESM.pdf]
